# A novel risk score model based on eight genes and a nomogram for predicting overall survival of patients with osteosarcoma

**DOI:** 10.1186/s12885-020-06741-4

**Published:** 2020-05-24

**Authors:** Guangzhi Wu, Minglei Zhang

**Affiliations:** 1 Departments of Hand Surgery, The Third Hospital of Jilin University, Changchun, Jilin Province China; 2 Departments of Orthopedics, The Third Hospital of Jilin University, Changchun, Jilin Province China

**Keywords:** Osteosarcoma, Differentially expressed genes, Support vector machine, Risk score, Prognosis

## Abstract

**Background:**

This study aims to identify a predictive model to predict survival outcomes of osteosarcoma (OS) patients.

**Methods:**

A RNA sequencing dataset (the training set) and a microarray dataset (the validation set) were obtained from The Cancer Genome Atlas (TCGA) and Gene Expression Omnibus (GEO) database, respectively. Differentially expressed genes (DEGs) between metastatic and non-metastatic OS samples were identified in training set. Prognosis-related DEGs were screened and optimized by support vector machine (SVM) recursive feature elimination. A SVM classifier was built to classify metastatic and non-metastatic OS samples. Independent prognosic genes were extracted by multivariate regression analysis to build a risk score model followed by performance evaluation in two datasets by Kaplan-Meier (KM) analysis. Independent clinical prognostic indicators were identified followed by nomogram analysis. Finally, functional analyses of survival-related genes were conducted.

**Result:**

Totally, 345 DEGs and 45 prognosis-related genes were screened. A SVM classifier could distinguish metastatic and non-metastatic OS samples. An eight-gene signature was an independent prognostic marker and used for constructing a risk score model. The risk score model could separate OS samples into high and low risk groups in two datasets (training set: log-rank *p* < 0.01, C-index = 0.805; validation set: log-rank *p* < 0.01, C-index = 0.797). Tumor metastasis and RS model status were independent prognostic factors and nomogram model exhibited accurate survival prediction for OS. Additionally, functional analyses of survival-related genes indicated they were closely associated with immune responses and cytokine-cytokine receptor interaction pathway.

**Conclusion:**

An eight-gene predictive model and nomogram were developed to predict OS prognosis.

## Background

Osteosarcoma (OS) is a common malignant bone cancer in adolescents around the whole world and has a high tendency of metastasis [1]. The considerable progress in OS prevention and treatment has been made by introducing promising therapeutic strategies such as postoperative neo-adjuvant chemotherapy and multi-agent systemic chemotherapy over the past decades [2, 3]. However, the statistical evidence suggested that the incidence and mortality rates of OS have been continuously growing by approximately 1.4% each year [4]. The recent studies demonstrated that the 5-year survival rate of OS remains about 65% and more than half of OS patients die from OS metastasis [5, 6]. Therefore, the identification of novel prognostic gene markers for metastasis of OS is imperative for improving the overall survival of OS patients.

The advent of next sequencing technologies allows rapid disease detection and diagnosis in recent decades. Accordingly, extensive studies based on the microarray data and transcriptome sequencing data were carried out to identify potential gene drivers involved in the occurrence, metastasis and recurrence of tumors. Notably, existing evidence has showed that numerous gene signatures had significant prognostic values for OS. Wang et al argued that OS patients with a high *ALDH1B1* level had an unfavorable clinical outcome compared to those OS patients with low *ALDH1B1* level, implying that this gene might be a potential prognostic marker for patients with OS [7]. Shi et al examined the expression difference and prognostic power of *DDX10* based on a dataset from Gene Expression Omnibus (GEO) database. They found that there was a higher expression level of *DDX10* in OS tissues than normal tissues and increased *DDX10* level was related to a poor prognosis [8]. Furthermore, a previous research analyzed three miRNA expression profiles and constructed a support vetor mechine (SVM) classifier with 15 differentially expressed miRNAs (DEmiRNAs). The results showed that this classifier had a relatively high accuracy to predict OS recurrence, suggesting that these DEmiRNAs were possibly associated with OS prognosis [9]. Liu et al recently screened a four-pseudogene signature for OS survival prediction based on the RNA sequencing data and this pseudogene panel could clearly differentiate high and low risk patients with OS [10]. Although previous studies have identified many gene makers in the development and recurrence, a deeper understanding of the influence of gene signatures on the survival prognosis of OS needs further investigating.

In the present research, the differentially expressed genes (DEGs) between metastatic and non-metastatic OS samples were identified from the training dataset obtained from The Cancer Genome Atlas (TCGA) database. Then, the prognostic genes were screened followed by optimized selection based on SVM recursive feature elimination (SVM-RFE) algorithm. The optimal prognostic genes were used to construct a SVM classifier to separate OS metastatic and non-metastatic OS samples. Additionally, a machine learning analysis (univariate and multivariate cox regression) was used to extract independent OS prognostic genes to construct a risk score (RS) model. The independent clinical prognostic indicator was identified followed by a predictive nomogram construction. Finally, the functional analyses of prognosis-related genes were also performed. Our findings will promote the understanding of clinical prognostic outcomes of OS patients.

## Methods

### Data source

The level 3 mRNA sequencing data of OS patients was firstly downloaded from TCGA (https://gdc-portal.nci.nih.gov/) database, which was generated by Illumina HiSeq 2000 RNA sequencing platform and concluded 265 samples. Subsequently, we mapped these samples into their clinical characteristics obtained and removed those samples without metastasis information. Finally, a total of 176 OS samples (58 metastatic samples and 118 non-metastatic samples) were included in this study. Accordingly, the mRNA sequencing data from these samples and corresponding clinical information were retained and considered as the training dataset in following analysis. In addition, the OS gene expression matrix from GSE21257 dataset provided by Buddingh et al [11] was acquired from GEO [12] repository (http://www.ncbi.nlm.nih.gov/geo/). This dataset was based on Illumina human-6 v2.0 expression beadchip platform and comprised of 53 OS samples (34 samples with OS metastasis and 19 samples without OS metastasis). Moreover, these samples all had the relevant clinical prognosis information, and therefore, we regarded this dataset as the validation dataset.

### Identification of differentially expressed genes (DEGs)

For the training dataset, all samples were divided into two groups (metastatic and non-metastatic group). Then, we employed the limma [13] package (version 3.34.7; https://bioconductor.org/packages/release/bioc/html/limma.html) in R 3.4.1 to identify the significantly DEGs between OS metastasis samples and OS without metastasis samples according to the thresholds of false discovery rate (FDR) < 0.05 and the |log_2_ fold change (FC)| > 0.5. Furthermore, the bidirectional hierarchical clustering analysis of DEGs in training dataset was carried out based on the centered pearson correlation algorithm using the pheatmap [14] package (version 1.0.8; https://cran.r-project.org/web/packages/pheatmap/index.html) in R 3.4.1.

### Screening of prognostic genes for OS

Firstly, the univariate cox regression analysis was performed to extract key genes associated with overall survival using the survival [15] package (version 2.41.1; http://bioconductor.org/packages/survivalr/) in R 3.4.1. The log-rank *p* value < 0.05 was thought of as the cutoff for the significant correlation. Afterwards, the e1071 [16] package (version 1.7.1; https://cran.r-project.org/web/packages/e1071) and caret [17] package (version 6.0.76; https://cran.r-project.org/web/packages/caret) in R 3.4.1 were utilized to further screen the optimal prognostic gene set (OPGS) for OS on the basis of SVM-RFE method, which is an iterative backward selection algorithm and can recursively remove one feature gene with the smallest ranking score until the optimal feature gene set was remained [18]. Following this, the SVM classifier was constructed for predicting OS metastasis based on the expression levels of OPGS. Moreover, the external GSE21257 dataset was used to verify the results of SVM classification analysis. The partial receiver operating characteristic (pROC) [19] package (version 1.15.0; https://cran.r-project.org/web/packages/pROC/index.html) was utilized to conduct performance evaluation of SVM classifier in training and validation dataset in R 3.4.1. Accordingly, the multiple quantization parameters in classification assessment task were computed, including sensitivity, specificity, area under curve (AUC), positive predictive value (PPV) and negative predictive value (NPV).

### Construction of prognostic model for OS and performance evaluation

The multivariate cox regression analysis was carried out to extract independent prognostic genes for OS using survival package in R 3.4.1 according to the cutoff criterion of the log-rank *p* value < 0.05. Afterwards, a risk score model of the prognostic mRNA makers was established according to following formula: Risk score (RS) = ∑β_DEGs_ × Exp_DEGs._ The β_DEGs_ represented the estimated contribution coefficient of independent prognostic mRNAs in the multivariate Cox regression analysis and Exp_DEGs_ denoted the level of independent prognostic genes. According to this formula, the RS of each OS patient was computed. Then, all patients in training dataset were divided into high and low risk groups with the median of the RS as the cut-off criterion. Furthermore, the survival differences between these two risk groups were evaluated by a Kaplan-Meier (KM) survival curve using the survival package in R 3.4.1. Meanwhile, the expression values of independent prognostic genes were also extracted from validation set and the RS for each sample was then calculated. Accordingly, the samples in validation dataset were also divided into two groups (high-risk and low-risk groups) based on the same strategy above mentioned. Finally, the difference in survival prognosis between the high-and low-risk group was also assessed according to three indicators (Harrell C-index, Brier score and log-rank *p* value of cox proportional hazards regression) [20–22]. Specifically, Harrell C-index, and Brier score were computed by the survcomp [23] package (version 1.30.0; http://www.bioconductor.org/packages/release/bioc/html/survcomp.html) in R 3.4.1. The R survival package was used to undertake the KM estimates of survival probability and calculate the corresponding log-rank *p* values [15].

### Screening of independent prognostic factors

The univariate and multivariate cox regression analyses were employed to identify the independent clinical prognostic factors using the survival package in R 3.4.1 with log-rank *p* value < 0.05 as the threshold for significance [15]. Following this, we further explored the relationships between independent prognostic factors and survival prognosis. A nomogram was constructed based on independent prognosis-related genes and prognostic factors to predict survival rates of patients at 3 and 5 years via the rms package (version 5.1.2; https://cran.r-project.org/web/packages/rms/index.html) in R 3.4.1 [22, 24].

### Functional analyses of key genes in high and low risk groups

The samples from training dataset were divided into high-risk and low-risk groups according to the RS in prognostic model. We then used R limma package to identify the DEGs between these two groups according to FDR < 0.05 and |log_2_FC| > 0.5 [13]. Subsequently, the Gene Ontology (GO)-biological process (BP) analysis and the Kyoto Encyclopedia of Genes and Genomes (KEGG) pathway enrichment analysis of these genes were carried out using the clusterProfiler [25] package (version 3.6.0; http://bioconductor.org/packages/release/bioc/html/clusterProfiler.html) in R 3.4.1. The FDR < 0.05 was considered as the threshold for significant enrichment.

## Results

### DEGs identification

**Fig. 1:**
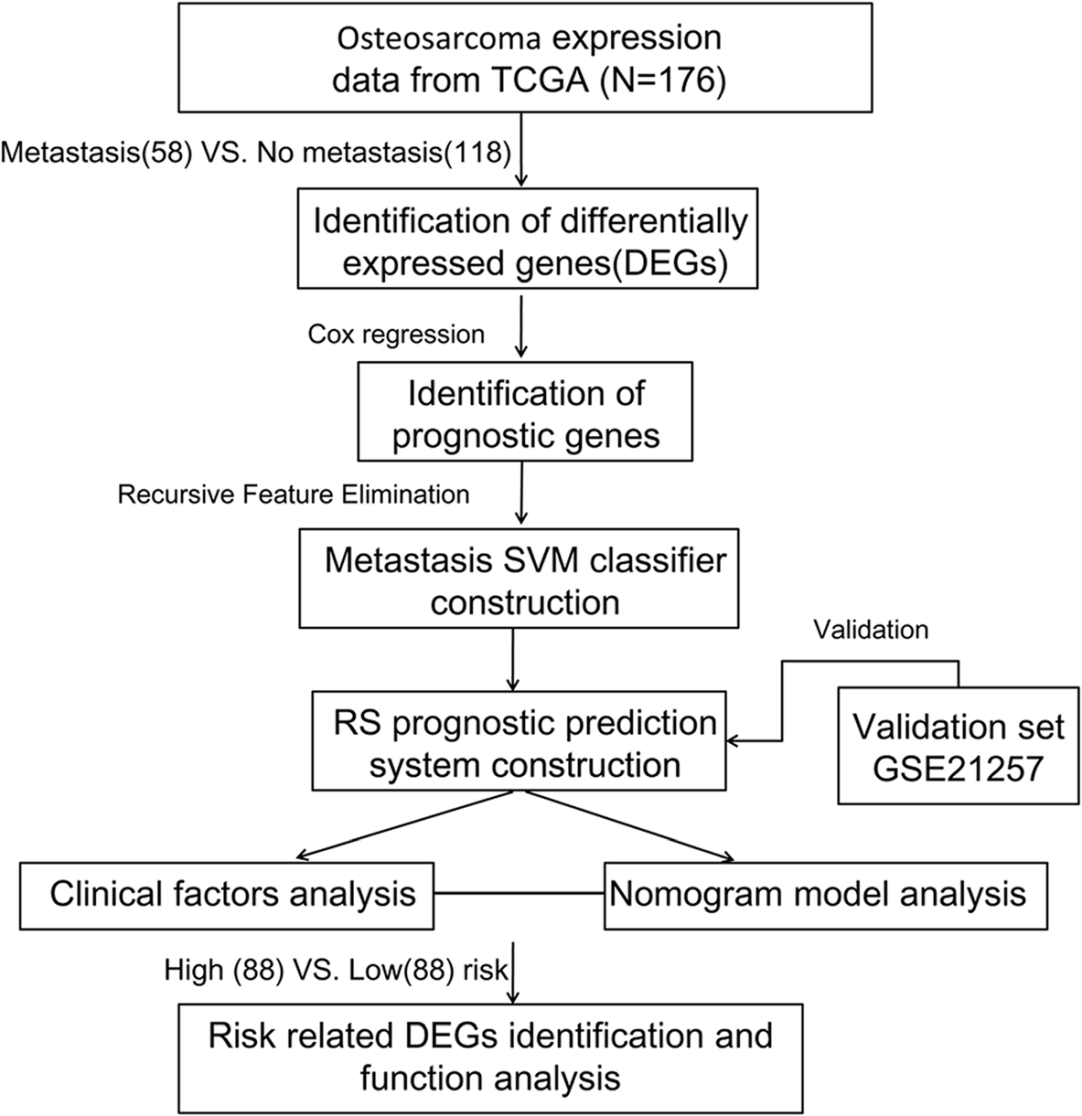
The flow chart of the whole analysis in this study

**Fig. 2:**
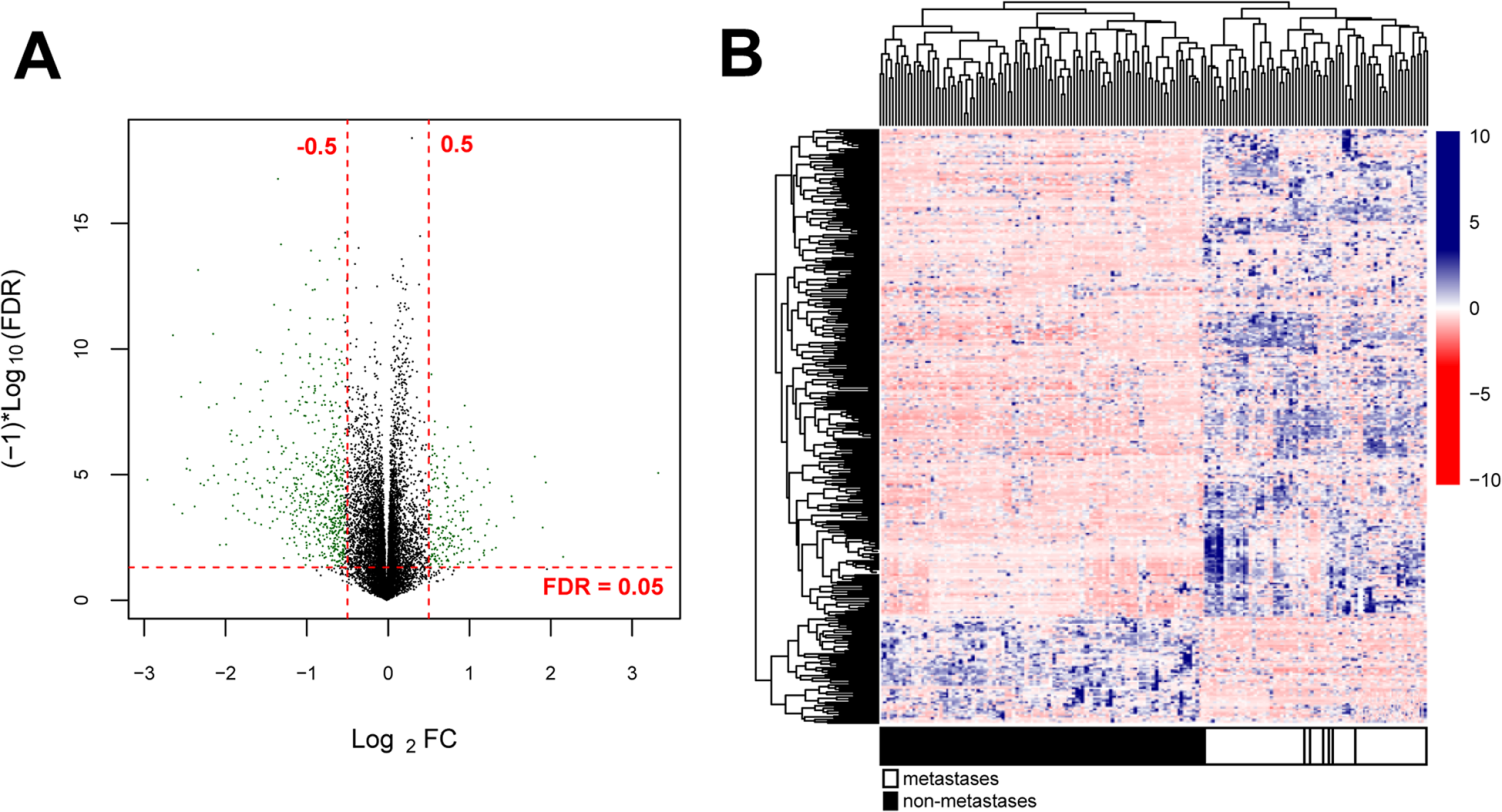
Volcano plot and heatmap clustering of differentially expressed genes (DEGs). **a**: Volcano plot of DEGs. The green nodes represent DEGs; the red horizontal dashed lines show the false discovery rate (FDR) value is less than 0.05 and the red vertical dashed lines indicate the value of |log_2_ fold change (FC)| is more than 0.5. **b**: Heatmap clustering of DEGs. The white bars represent metastatic osteosarcoma samples and black bars represent non-metastatic osteosarcoma samples

This study was carried out as showed in Fig. 1. In total, 345 DEGs were identified between metastatic and non-metastatic OS samples in training group, which contained 48 up-regulated genes and 297 down-regulated genes (Table S1). The volcano plot of these DEGs was displayed in Fig. 2a. In addition, the bidirectional hierarchical clustering analysis was carried out based on the expression profiles of these DEGs. All samples were divided into two groups (metastasis and non-metastasis). We found that 58 metastatic OS samples were all grouped into metastatic cluster. Meanwhile, most of non-metastatic OS samples (111/118) were clustered and seven non-metastatic OS samples were misclassified into metastatic cluster. The accuracy of metastasis identification was 96%, suggesting that these DEGs exhibited a good discriminative ability for differentiating the metastatic and non-metastatic OS samples (Fig. 2b).

### Screening of prognostic feature genes for OS

**Fig. 3:**
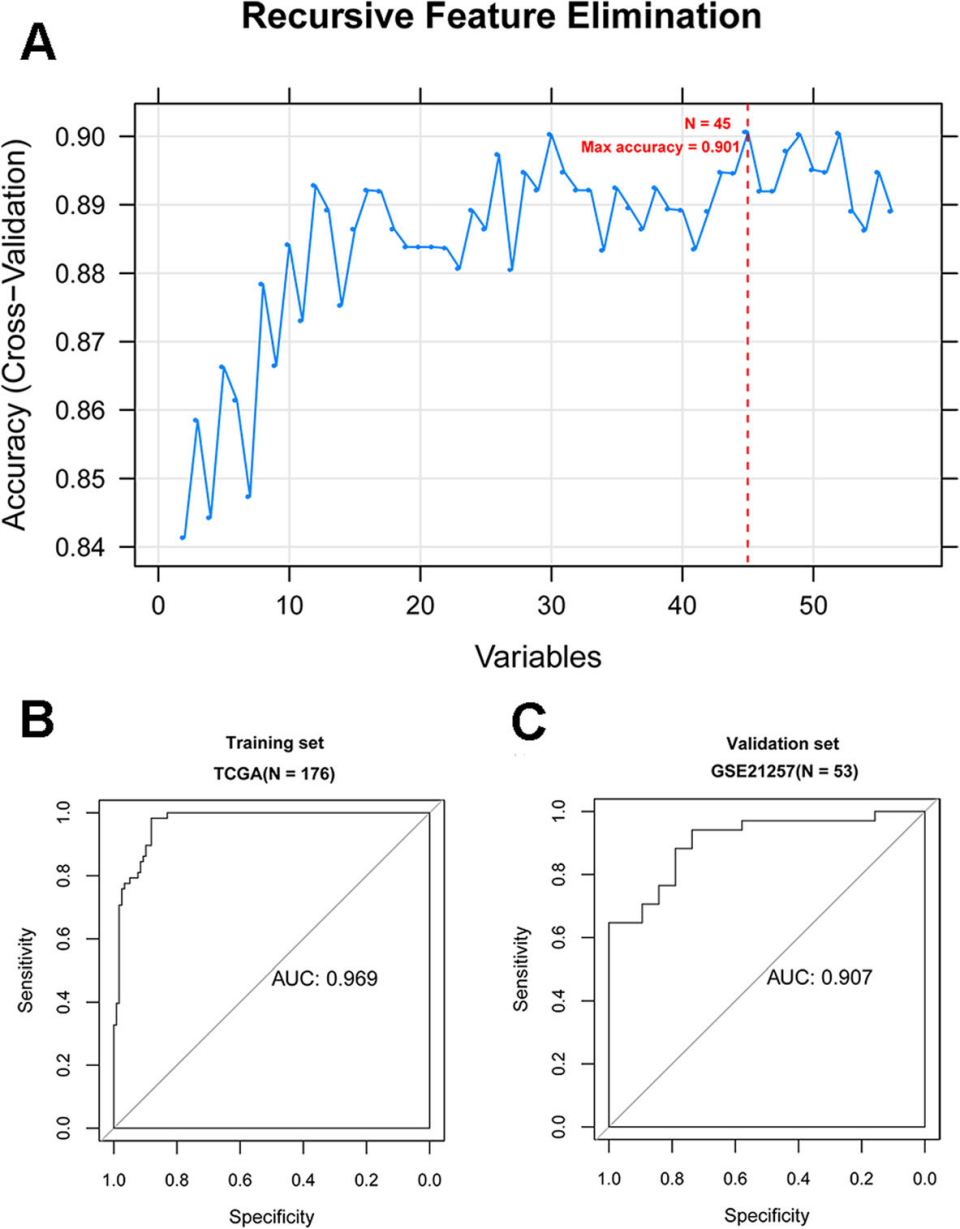
Screening of the optimal prognostic gene set for osteosarcoma and the receiver operating characteristic (ROC) curve of SVM classification. **a**: The identification of optimal prognostic gene set for osteosarcoma based on the recursive feature elimination algorithm. The horizontal axis shows the number of differentially expressed genes and the vertical axis represents the cross-validation accuracy. **b**: The ROC curve of SVM classification in training dataset. **c**: The ROC curve of SVM classification in validation dataset

The univariate cox regression analysis was performed to identify prognostic genes, and the results revealed that 56 DEGs were significantly correlated with overall survival of patients with OS. To obtain the most representative prognostic genes for metastatic OS, the SVM-RFE method was used to extract the OPGS. Consequently, a total of 45 DEGs were representatively prognosis-related genes with maximum accuracy of 0.901 as showed in Fig. 3a. Furthermore, a SVM-based classifier was constructed on the basis of these 45 DEGs according to the strategy described in method. The performance assessment analysis of the SVM classifier indicated that it could effectively distinguish metastatic OS from non-metastatic OS samples in training dataset with multiple evaluation indicators (the AUC of 0.969, the sensitivity of 0.915, the specificity of 0.884, the PPV of 0.741 and the NPV of 0.966; Table 1; Fig. 3a and b). Similarly, this classifier also exhibited a good discrimination between metastatic and non-metastatic OS samples in validation dataset based on the relatively high values of evaluation index (the AUC of 0.907, the sensitivity of 0.857, the specificity of 0.778, the PPV of 0.882 and the NPV of 0.737; Table 1; Fig. 3a and b). These results suggested that this OPGS was predominately associated with the survival outcomes for OS patients and the SVM classifier based on OPGS had a clinical implication for OS metastasis diagnosis.

**Table 1:** The performance evaluation of a SVM classifier in training and validation dataset

	ROC				
Datasets	AUROC	Sensitivity	Specificity	PPV	NPV
Training set (TCGA, *N* = 176)	0.969	0.915	0.884	0.741	0.966
Validation set (GSE21257, *N* = 53)	0.907	0.857	0.778	0.882	0.737

*SVM* Support vector machine, *ROC* Receiver operating characteristic, *AUROC* Area under the receiver operating characteristic curve, *PPV* Positive predictive value, *NPV* Negative predictive value

### Screening of independent prognostic feature DEGs and risk model construction

**Fig. 4:**
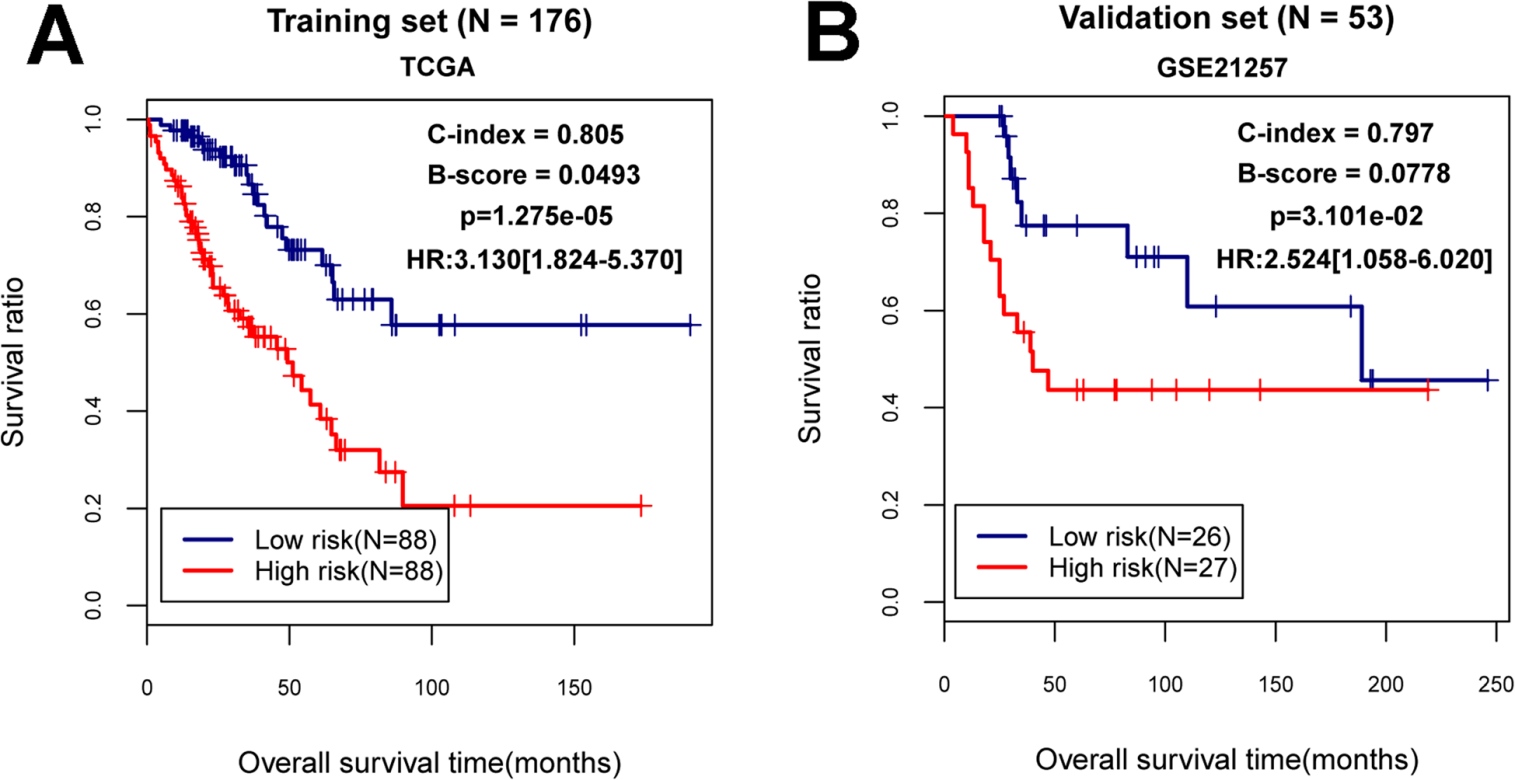
Kaplan–Meier survival analysis in training and validation sets. **a**: The KM curve based on RS and survival outcomes in training dataset. **b**: The KM curve based on RS and survival outcomes in validation dataset. HR: hazard ratio; C-index: Harrell concordance index; B-score: Brier score. The red and blue lines respectively represent high risk samples and low risk samples

The multivariate cox regression analysis was conducted to obtain the independent prognostic feature genes. As shown in Table 2, eight genes were found to be independently related to OS prognosis, including *KCNJ15* (potassium voltage-gated channel subfamily J member 15), *SLC24A4* (solute carrier family 24 member 4), *ASPA* (aspartoacylase), *REM1* (RRAD and GEM like GTPase 1), *SCARA5* (scavenger receptor class A member 5), *LANCL3* (LanC like 3), *CPA6* (carboxypeptidase A6) and *TRH* (thyrotropin releasing hormone). Afterwards, the expression levels of these genes in training dataset were computed and RS prediction model was constructed as follows: RS = (0.0501) × Exp_KCNJ15_ + (− 0.392) × Exp_SLC24A4_ + (0.0661) × Exp_ASPA_ + (− 0.0633) × Exp_REM1_ + (− 0.024) × Exp_SCARA5_ + (0.143) × Exp_LANCL3_+ (0.0522) × Exp_CPA6_ + (0.0592) × Exp_TRH_. The RS for each sample was then calculated in training set. All samples (*n* = 176) were classified into high risk group (*n* = 88) and low risk group (*n* = 88) in the light of the median value of RS. The survival analysis revealed that there was a significant correlation between two different risk groups and survival outcomes (Hazard Ratio [HR] = 3.130, 95% Confidence intervals [CI]: 1.824–5.370, log-rank *p* = 1.275e-0.5, Harrell C-index = 0.805 and Brier score = 0.049; Fig. 4a). Meanwhile, the samples (*n* = 53) in validation set were also stratified into high-risk group (*n* = 27) and low-risk group (*n* = 26) using the same method. The dramatic relationship was observed between these risk groups and clinical survival in validation set (HR = 2.524, 95% CI: 1.058–6.020, log-rank *p* = 3.101e-02, Harrell C-index = 0.797 and Brier score = 0.078; Fig. 4b). Notably, we found that the patients from low risk group had higher survival probabilities than those patients from high risk group in both training and validation set (Fig. 4).

**Table 2:** The list of independent prognostic feature genes

Gene	coef	***P*** value	Hazard Ratio	95%CI
KCNJ15	0.0501	2.220E-03	1.0513	1.0182–1.0856
SLC24A4	− 0.392	4.840E-03	0.6757	0.5145–0.8875
ASPA	0.0661	7.220E-03	1.0683	1.0180–1.1211
REM1	−0.0633	2.152E-02	0.9386	0.8893–0.9907
SCARA5	−0.024	2.287E-02	0.9763	0.9564–0.9967
LANCL3	0.143	3.370E-02	1.1533	1.0111–1.3155
CPA6	0.0522	3.787E-02	1.0536	1.0029–1.1067
TRH	0.0592	4.298E-02	1.0610	1.0019–1.1236

*Coef* Coefficient derived from multiivariate cox regression analysis, *95%CI* 95% confidence interval

### Predictive nomogram model of independent prognostic factors

**Fig. 5:**
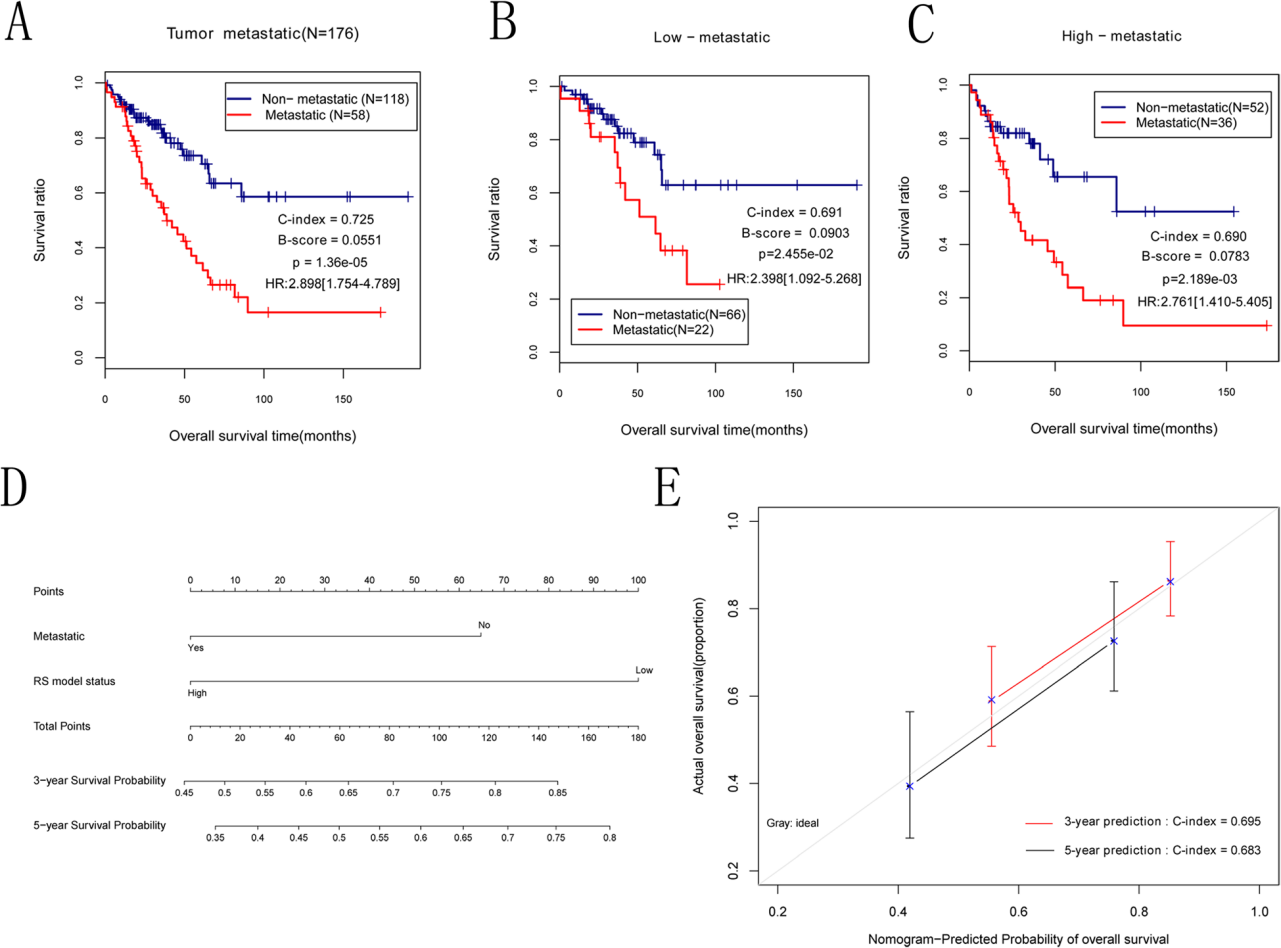
Kaplan–Meier survival analysis and prognostic nomogram model. **a**: Kaplan-Meier curve comparing the survival rate between patients with and without osteosarcoma metastasis in TCGA cohort. **b**: Kaplan-Meier curve comparing the survival rate between patients with and without tumor metastasis from high risk group in TCGA cohort. **c**: Kaplan-Meier curve comparing the survival rate between patients with and without tumor metastasis from low risk group in TCGA cohort. The blue and red curves respectively represent metastatic osteosarcoma and non-metastatic osteosarcoma samples. **d**: The nomogram prediction for overall survival probability at 3- and 5-year for osteosarcoma patients in TCGA cohort. **e**: The calibration curve of nomogram to predict the probability of overall survival at 3, and 5 years for osteosarcoma patients in TCGA cohort; the X axis represents the predicted actual overall survival while the Y axis represents the actual overall survival. *TCGA* The Cancer Genome Atlas

To identify the independent prognostic indicators for OS survival, the univariate and multivariate regression analysis was performed based on the clinical features from of patients in training set. As indicated in Table 3, two independent prognostic parameters (tumor metastasis and RS model status) were remarkably linked with the OS clinical outcomes. Moreover, survival analysis showed that tumor metastasis was markedly related to overall survival (log-rank *p* = 1.36e-05 and HR = 2.898, 95% CI: 1.754–4.789) and patients with OS metastasis had a worse prognosis than those with OS non-metastasis, which was in accordance with clinical practice (Fig. 5a). Besides, there were also strong correlations between tumor metastasis and overall survival time in high and low risk groups (for high risk group: log-rank *p* = 2.455e-02 and HR = 2.398, 95% CI: 1.092–5.268; for low risk group: log-rank *p* = 2.189e-03 and HR = 2.761, 95% CI: 1.410–5.405; Fig. 5b and c). Subsequently, the tumor metastasis and RS model status were incorporated into a nomogram model for predicting the rates of overall survival at the 3- and 5-year in OS patients. The score of every indicator can be found by points scale located at the top of nomogram. Then, the points of each indicator were summed, thereby estimating survival probability at 3- and 5-year (Fig. 5d). Furthermore, we constructed a calibration curve to evaluate the performance of nomogram model. Notably, the C-index was respectively 0.695 and 0.683 for OS prediction at the 3- and 5-year, suggesting that the nomogram prediction for survival rates was in line with the actual observation for OS patients (Fig. 5e). These results demonstrated that the nomogram based on OS metastasis and RS model status exhibited a good predictive accuracy for survival prognosis of OS patients.

**Table 3:** The univariables and multi-variables cox regression of clinical parameters and survival outcomes of patients with osteosarcoma

Clinical characteristics	TCGA(***N*** = 176)	Uni-variables cox			Multi-variables cox		
		HR	95%CI	***P***	HR	95%CI	***P***
Age(years, mean ± sd)	61.01 ± 15.23	1.018	0.999–1.036	5.19E-02	–	–	–
Gender(Male/Female)	72/104	1.102	0.666–1.827	7.05E-01	–	–	–
Pathologic tumor depth(years, mean ± sd)	6.35 ± 3.69	1.136	0.951–1.227	9.05E-02	–	–	–
Pathologic tumor length(years, mean ± sd)	11.76 ± 7.25	1.061	0.993–1.092	5.91E-02	–	–	–
Pathologic tumor width(years, mean ± sd)	8.78 ± 5.51	1.091	0.940–1.143	2.17E-01	–	–	–
Tumor multifocal(Yes/No/−)	34/132/10	1.685	0.949–2.991	7.13E-02	–	–	–
Tumor recurrence(Yes/No/−)	28/141/7	2.692	1.581–4.585	1.48E-04	1.626	0.914–2.893	9.80E-02
Tumor metastatic(Yes/No/−)	58/118	2.898	1.754–4.789	1.36E-05	1.879	1.079–3.274	2.58E-02
Radiotherapy(Yes/No/−)	64/110/2	0.799	0.474–1.347	3.99E-01	–	–	–
Tumor necrosis(No/Slight/Moderate/Severe/−)	61/35/59/10/11	1.191	0.925–1.530	1.75E-01	–	–	–
RS model status(High/ Low)	88/88	3.13	1.824–5.370	1.28E-05	2.288	1.291–4.057	4.60E-03
Dead(Death/Alive/−)	63/113	–	–	–	–	–	–
Overall survival time(months, mean ± sd)	39.76 ± 32.24	–	–	–	–	–	–

*SD* Standard deviation, *HR* Hazard ratio, *95%CI* 95% confidence interval,  *TCGA*The Cancer Genome Atlas

### Screening of prognosis risk-related genes and functional analyses

**Fig. 6:**
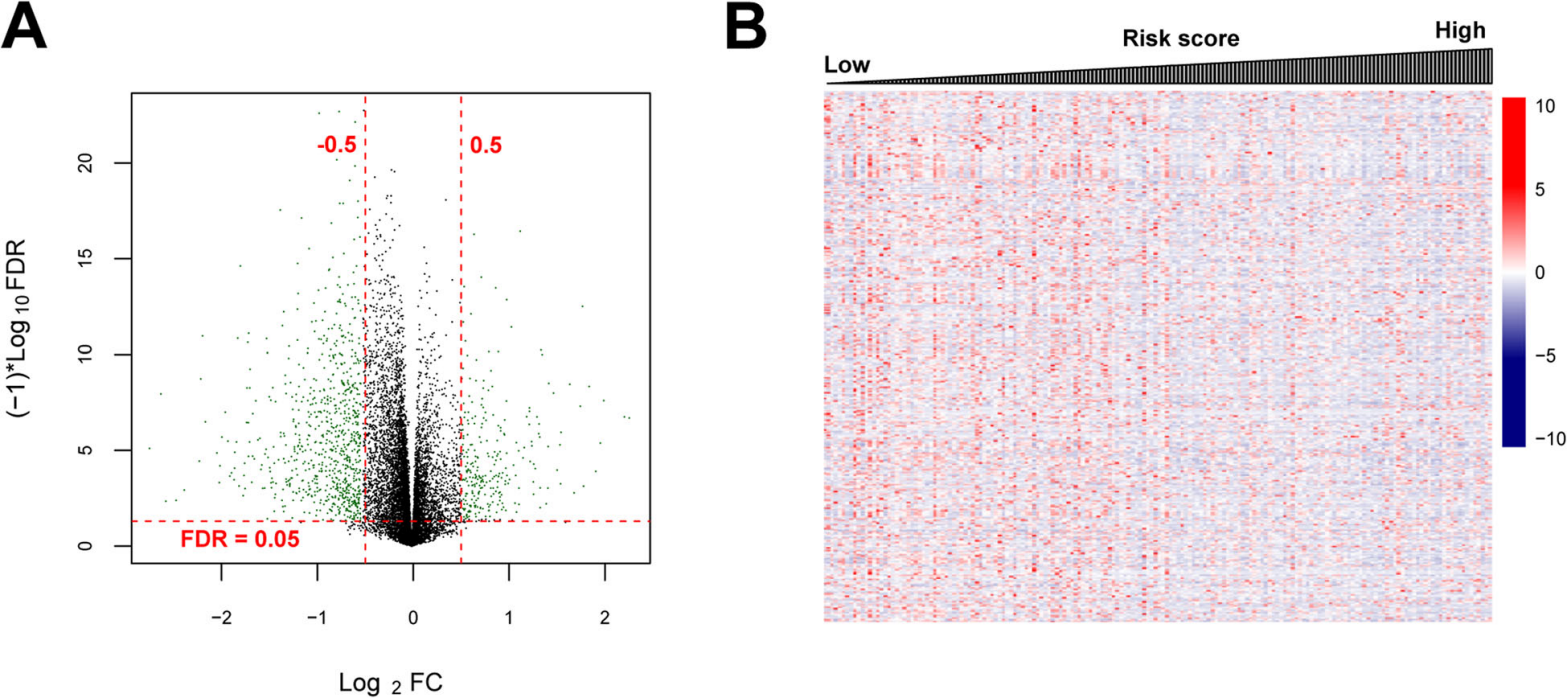
The differentially expressed genes (DEGs) between high and low risk groups in TCGA cohort. **a**: Volcano plot of DEGs. The green nodes represent DEGs and black color showed non-DEGs. **b**: The heatmap of DEGs. The expression changes from low to high expression levels with risk score. *TCGA* The Cancer Genome Atlas

The samples from training set were separated into high and low risk groups on the basis of RS model. The 614 DEGs were extracted between these two groups using the limma package in R 3.4.1, which comprised of 117 significantly up-regulated genes and 497 significantly down-regulated genes (Table S2). The volcano plot and heatmap of gene expression were showed in Fig. 6 a and b. Furthermore, the GO-BP annotation analysis and KEGG pathway analysis were undertaken for these DEGs. Our results indicated that these genes primarily played essential roles in 23 GO-BP terms including immune responses. Meanwhile, they were enriched in 8 KEGG pathways such as cytokine-cytokine receptor interaction pathway (Table 4).

**Table 4:** The functional analyses of survival-related genes

Category	Term	Count	***P*** value	FDR
GO-BP	GO:0002250~adaptive immune response	28	6.430E-13	1.560E-09
	GO:0050776~regulation of immune response	28	5.700E-11	6.900E-08
	GO:0070098~chemokine-mediated signaling pathway	18	1.450E-10	1.170E-07
	GO:0006955~immune response	41	3.820E-09	1.850E-06
	GO:0042110~T cell activation	14	3.170E-09	1.920E-06
	GO:0006935~chemotaxis	19	1.430E-07	5.750E-05
	GO:0006954~inflammatory response	35	2.300E-07	7.950E-05
	GO:0006968~cellular defense response	13	9.040E-07	2.730E-04
	GO:0007267~cell-cell signaling	26	1.760E-06	4.740E-04
	GO:0007166~cell surface receptor signaling pathway	27	2.180E-06	5.280E-04
	GO:0071346~cellular response to interferon-gamma	11	1.840E-05	4.037E-03
	GO:0042102~positive regulation of T cell proliferation	11	2.930E-05	5.894E-03
	GO:0007204~positive regulation of cytosolic calcium ion concentration	16	4.730E-05	8.769E-03
	GO:0035589~G-protein coupled purinergic nucleotide receptor signaling pathway	6	6.700E-05	1.152E-02
	GO:0043547~positive regulation of GTPase activity	38	9.630E-05	1.446E-02
	GO:0070374~positive regulation of ERK1 and ERK2 cascade	18	9.260E-05	1.484E-02
	GO:0007155~cell adhesion	32	2.060E-04	2.894E-02
	GO:0007165~signal transduction	63	2.210E-04	2.926E-02
	GO:0048247~lymphocyte chemotaxis	7	2.880E-04	3.606E-02
	GO:0042472~inner ear morphogenesis	9	3.260E-04	3.873E-02
	GO:0050850~positive regulation of calcium-mediated signaling	6	4.390E-04	4.712E-02
	GO:0006508~proteolysis	33	4.240E-04	4.774E-02
	GO:0002548~monocyte chemotaxis	8	4.660E-04	4.781E-02
KEGG Pathway	hsa04060:Cytokine-cytokine receptor interaction	38	4.250E-12	9.690E-10
	hsa04080:Neuroactive ligand-receptor interaction	34	4.100E-08	4.680E-06
	hsa04640:Hematopoietic cell lineage	17	4.960E-07	3.770E-05
	hsa04514:Cell adhesion molecules (CAMs)	19	2.440E-05	1.389E-03
	hsa04062:Chemokine signaling pathway	21	9.560E-05	4.350E-03
	hsa04660:T cell receptor signaling pathway	13	9.200E-04	2.590E-02
	hsa05033:Nicotine addiction	8	1.213E-03	3.028E-02
	hsa04650:Natural killer cell mediated cytotoxicity	14	1.739E-03	3.543E-02

*KEGG* Kyoto Encyclopedia of Genes and Genomes, *FDR* false discovery rate,  *GO-BP* Gene Ontology-Biology Process

## Discussion

Existing evidence has demonstrated that it is crucial to identify several key gene makers related to OS survival prognosis, which provides important theoretical references for developing promising therapeutic strategies for OS treatment. Herein, we established a SVM-based classifier to distinguish metastatic OS samples and non-metastatic OS samples. Moreover, eight independent prognostic genes were identified to construct a RS model. Meanwhile, tumor metastasis and RS model status were found to serve as independent prognostic factors for OS survival. Additionally, the functional analyses of prognosis-related genes reveled that they were significantly enriched in the GO-BP term of immune responses and cytokine-cytokine receptor interaction pathway.

Tumor metastasis is a leading cause of high mortality rates of various tumors. In recent decades, the high-throughput sequencing technologies have greatly facilitated the understanding of metastasis-related genes function by decoding the genome of cancer patients [26]. An increasing number of researchers have also concentrated on exploring the underlying pathogenesis of OS metastasis [27, 28]. Moreover, building a prediction model of OS metastasis was growingly important for prognostication and clinical decision-making. A SVM, which could effectively distinguish entities into different classes in analyzing microarray data, was frequently used in constructing sample classification model due to its high accuracy and flexibility for modeling multisource data [29]. He et al established a SVM classifier using 64 feature genes for OS and this classifier differentiated metastatic OS samples from non-metastatic OS samples in the dataset GSE21257 with a prediction accuracy of 100% [30]. Herein, we constructed a SVM classifier based on 45 prognosis-related genes to discriminate OS metastasis samples and non-metastasis samples. The performance evaluation analysis revealed that this classifier had high precision with AUC of 0.969, sensitivity of 0.915 and specificity of 0.884. Moreover, these results generated in training set were also verified by a SVM classification in validation set GSE21257. The SVM classifier also exhibited a good performance with AUC of 0.907, sensitivity of 0.857 and specificity of 0.778. These findings implied that 45 prognostic genes might be key biomarkers to identify metastatic and non-metastatic OS patients.

The correlations between these prognosis-related genes and clinical survival were investigated by a multivariate logistic regression analysis. The results showed that there were eight independent prognostic risk genes for OS, which consisted of *KCNJ15*, *SLC24A4*, *ASPA*, *REM1*, *SCARA5*, *LANCL3*, *CPA6* and *TRH*. A RS model was also constructed to divide OS patients into high and low-risk groups. Consequently, this eight-gene signature exhibited a good performance to differentiate metastatic OS patients from non-metastatic OS patients. *KCNJ15* (also known as *KIR4.2*), belongs to a member of the *KIR4* subfamily and encodes the potassium channel. Liu et al recently reported the silencing of *KCNJ15* played key roles in tumor malignance and was related to unfavourable prognosis renal carcinoma [31]. However, whether *KCNJ15* directly involves in OS progression has not been clarified. Notably, multiple investigations have demonstrated that some potassium ion channels such as hSlo potassium channel in OS cells were implicated with the carcinogenesis [32, 33]. Therefore, the potential roles of *KCNJ15* in pathogenesis of OS need to be investigated in future. *SLC24A4*/NCKX4 is located at on chromosome region 14q32 and a member of potassium-dependent sodium-calcium exchanger gene family [34]. No reports are concerned about the relationship of this gene and OS development. *REM1* encodes a GTPase and participates regulating the activity of voltage-dependent Ca^2+^ channels. Numerous studies have suggested that *SCARA5*, a member of the scavenger receptor family, is involved in the molecular mechanisms of various cancers such as hepatocellular carcinoma [35]. You et al previously found that *SCARA5* served as a key biomarker for the development and metastasis of breast cancer [36]. An early research reported that *CPA6* was remarkably up-regulated in early stage samples with oral squamous cell carcinoma (OSCC) compared with those in late stage, suggesting that this gene might have crucial diagnostic values for OSCC [37]. Another study emphasized that the methylation of *TRH* could classify OSCC and oropharyngeal SCC patients from healthy individuals with a high accuracy [38]. Unfortunately, the association of eight independent prognostic genes and OS has not been unraveled until now. Further investigations are essential to understand the underlying role of these genes for the diagnosis and prediction of OS.

Additionally, the DEGs between high and low risk groups in training dataset were further extracted to understand the influence of this eight-gene signature on OS prognosis prediction. There were 614 significantly DEGs (117 up-regulated and 497 down-regulated genes). The GO-BP analysis indicated that these genes were mainly responsible for immune responses. Mori et al argued that up-regulation of the immune response was a critical characteristic in patients with tumors and several immunotherapies were potent approaches for those patients undergoing OS metastasis [39]. Moreover, a wealth of evidence has suggested that the involvement of immunotherapies could regulate the tumor microenvironment and re-activate prolonged immune responses [40, 41]. The results of KEGG enrichment analysis showed that these genes were predominately associated with cytokine-cytokine receptor interaction pathway. Similarly, Chen et al performed a bioinformatics analysis based on a circRNA microarray dataset and three gene expression profiles of OS cell lines. They found that those down-regulated DEGs from three gene profiles mainly played prominent roles in cytokine-cytokine receptor interaction pathway [42]. These findings revealed that the initiation of immune responses and cytokine-cytokine receptor interaction possibly contributed to the OS progression.

In the current study, we also found tumor metastasis and RS model status acted as independent prognostic factors for OS survival by the cox regression model analysis. Then, these two survival-related factors as variables were incorporated into the nomogram and the results indicated that RS model status showed the biggest influence on OS survival prognosis. The nomogram is a powerful risk assessment tool for a wide variety of diseases including OS, which provides important guidance for clinical outcomes prediction, therapy selection and follow-up care [43]. We noted that overall survival rates the 3- and 5-year for OS patients were similar to the actual observation for OS patients, implying that tumor metastasis and RS model status were vital clinical characteristics for survival prediction of OS patients.

Although an eight-gene panel and two independent prognostic factors have been identified to be associated with OS prognosis, the detailed pathologic mechanisms have not been elucidated. For example, whether these gene signatures are involved in several molecular pathways such as cytokine-cytokine receptor interaction pathway still needs to be illuminated. Moreover, a further accurate classification with a large sample size and clinical information is necessary to distinguish OS metastasis and non-metastasis patients. In addition, the external validation is not carried out to check the reliability of our nomogram. Meanwhile, the performance evaluation of nomogram established here also requires to be performed. Finally, the corresponding experimental research is also needed to verify the biological functions of key gene.

In conclusion, we constructed a SVM-based classifier to separate metastatic and non-metastatic patients. Moreover, the eight-gene signature and two independent prognostic factors (tumor metastasis and RS model status) were closely related to OS survival. These findings greatly improved the understandings of OS metastasis and prognosis. However, relevant validation studies and optimization of prognostic model for OS will be considered in future.

## Conclusion

An eight-gene predictive model and nomogram were developed to predict OS prognosis.

## Supplementary information

**Additional file 1: Table S1**. The list of differentially expressed genes between metastatic and non-metastatic osteosarcoma patients in training dataset was displayed. **Table S2.** The list of differentially expressed genes between high and low risk groups in training dataset.

## Data Availability

The raw data were collected and analyzed by the Authors, and are not ready to share their data because the data have not been published.

## Abbreviations

OS: Osteosarcoma GEO Gene expression omnibus GO Gene Ontology
TCGA: The Cancer Genome Atlas
DEGs: Differentially expressed genes
SVM: Support vector machine
DEmiRNA: Differentially expressed miRNAs
SVM-RFE: SVM recursive feature elimination
RS: Risk score
FDR: False discovery rate
FC: Fold change
OPGS: Optimal prognostic gene set
pROC: Partial receiver operating characteristic
AUC: Area under curve
PPV: Positive predictive value
NPV: Negative predictive value
RS: Risk score
KM: Kaplan-Meier
BP: Biological process
KEGG: Kyoto Encyclopedia of Genes and Genomes
HR: Hazard Ratio
CI: Confidence intervals
OSCC: Oral squamous cell carcinoma
