# Supplementary material for: A novel risk score model based on eight genes and a nomogram for predicting overall survival of patients with osteosarcoma
Source: BMC Cancer. 2020 May 24;20:456. doi: 10.1186/s12885-020-06741-4 (PMC7245838; doi:10.1186/s12885-020-06741-4)
Supplement: Supplementary file 1 — Additional file 1: Table S1. The list of differentially expressed genes between metastatic and non-metastatic osteosarcoma patients in training dataset was displayed. Table S2. The list of differentially expressed genes between high and low risk groups in training dataset. [file 12885_2020_6741_MOESM1_ESM.docx]

**Table S1 The list of differentially expressed genes between metastatic and non-metastatic osteosarcoma patients in training dataset.**

| **Regulation** | **Gene Symbol** | **Log_2_FC** | ***P* value** | **FDR** |
| --- | --- | --- | --- | --- |
| *Up-regulation* | CAMK2N2 | 0.517 | 1.892E-04 | 2.892E-03 |
|  | BMPR1Bc | 0.526 | 1.244E-03 | 1.902E-02 |
|  | TNNT1 | 0.527 | 3.208E-03 | 4.904E-02 |
|  | TMEM108 | 0.550 | 1.293E-04 | 1.976E-03 |
|  | BTBD11 | 0.552 | 2.922E-03 | 4.467E-02 |
|  | LANCL3 | 0.555 | 1.787E-03 | 2.732E-02 |
|  | ZDHHC23 | 0.563 | 1.199E-03 | 1.833E-02 |
|  | KREMEN2 | 0.564 | 9.279E-04 | 1.419E-02 |
|  | FSD1 | 0.565 | 1.028E-03 | 1.572E-02 |
|  | COX8C | 0.569 | 2.353E-03 | 3.597E-02 |
|  | C17orf78 | 0.588 | 2.277E-03 | 3.481E-02 |
|  | FOXL1 | 0.590 | 1.147E-03 | 1.753E-02 |
|  | ITGA10 | 0.590 | 6.739E-04 | 1.030E-02 |
|  | KCNJ15 | 0.597 | 1.934E-03 | 2.957E-02 |
|  | CA3 | 0.603 | 2.854E-03 | 4.363E-02 |
|  | SGIP1 | 0.616 | 1.223E-03 | 1.870E-02 |
|  | HIST1H3B | 0.621 | 1.637E-03 | 2.502E-02 |
|  | HIST1H3G | 0.621 | 1.692E-03 | 2.587E-02 |
|  | TMEM74 | 0.625 | 2.264E-03 | 3.461E-02 |
|  | PAX9 | 0.693 | 2.003E-03 | 3.062E-02 |
|  | CKMT2c | 0.698 | 9.933E-04 | 1.518E-02 |
|  | GPR62 | 0.706 | 1.670E-03 | 2.554E-02 |
|  | LHFPL1 | 0.715 | 2.542E-03 | 3.887E-02 |
|  | LRFN5 | 0.716 | 1.376E-03 | 2.104E-02 |
|  | SH3RF2 | 0.728 | 2.637E-03 | 4.032E-02 |
|  | KCNQ5 | 0.736 | 2.226E-03 | 3.402E-02 |
|  | CREG2 | 0.738 | 3.234E-03 | 4.944E-02 |
|  | TNNI3K | 0.743 | 2.857E-03 | 4.368E-02 |
|  | MAPT | 0.749 | 5.518E-04 | 8.436E-03 |
|  | SLC44A5 | 0.751 | 2.826E-03 | 4.320E-02 |
|  | COL10A1 | 0.762 | 2.922E-03 | 4.467E-02 |
|  | FGF5 | 0.866 | 2.813E-03 | 4.301E-02 |
|  | KLF17 | 0.878 | 9.337E-04 | 1.427E-02 |
|  | PRPH | 0.907 | 1.491E-03 | 2.280E-02 |
|  | MMP13 | 0.940 | 1.967E-03 | 3.008E-02 |
|  | BBOX1 | 0.959 | 3.067E-04 | 4.688E-03 |
|  | IGF2BP1 | 0.990 | 2.278E-03 | 3.483E-02 |
|  | KCTD16 | 1.029 | 8.379E-04 | 1.281E-02 |
|  | RPL3L | 1.035 | 5.445E-04 | 8.324E-03 |
|  | SYNGR4 | 1.047 | 3.268E-03 | 4.996E-02 |
|  | WNT7B | 1.070 | 1.052E-03 | 1.609E-02 |
|  | HTR4 | 1.113 | 2.389E-03 | 3.652E-02 |
|  | ABCA13c | 1.195 | 2.902E-03 | 4.437E-02 |
|  | CPA6 | 1.244 | 1.763E-03 | 2.695E-02 |
|  | FNDC7 | 1.267 | 2.562E-03 | 3.917E-02 |
|  | NTSR1 | 1.822 | 1.248E-03 | 1.908E-02 |
|  | SERPINI2 | 1.957 | 2.549E-03 | 3.896E-02 |
|  | GAD2 | 3.340 | 1.954E-03 | 2.988E-02 |
| *Down-regulation* | XKR4 | -2.941 | 2.364E-03 | 3.614E-02 |
|  | LRRTM4 | -2.628 | 4.386E-05 | 6.705E-04 |
|  | IGLL1 | -2.526 | 2.397E-04 | 3.665E-03 |
|  | CAPN13 | -2.461 | 1.738E-03 | 2.657E-02 |
|  | RFX4 | -2.452 | 1.314E-03 | 2.009E-02 |
|  | DGKK | -2.418 | 1.814E-03 | 2.773E-02 |
|  | TDRD9 | -2.319 | 7.215E-06 | 1.103E-04 |
|  | OLIG1 | -2.290 | 1.619E-04 | 2.474E-03 |
|  | FCRL1 | -2.290 | 2.625E-03 | 4.014E-02 |
|  | HNF4A | -2.246 | 2.759E-03 | 4.218E-02 |
|  | CR2 | -2.196 | 2.379E-03 | 3.638E-02 |
|  | PROK1 | -2.187 | 3.218E-04 | 4.919E-03 |
|  | BLK | -2.134 | 4.229E-05 | 6.465E-04 |
|  | SV2C | -2.131 | 1.608E-03 | 2.459E-02 |
|  | FCRL2 | -2.123 | 2.089E-03 | 3.193E-02 |
|  | ACE2 | -2.088 | 2.938E-04 | 4.491E-03 |
|  | CA6 | -2.071 | 2.603E-03 | 3.979E-02 |
|  | FA2H | -1.947 | 2.052E-03 | 3.137E-02 |
|  | ONECUT1 | -1.926 | 6.683E-04 | 1.022E-02 |
|  | DLGAP2 | -1.909 | 8.158E-04 | 1.247E-02 |
|  | KCNJ6 | -1.908 | 6.062E-04 | 9.267E-03 |
|  | FGF20 | -1.873 | 2.479E-04 | 3.790E-03 |
|  | ATP6V0A4 | -1.860 | 2.371E-03 | 3.624E-02 |
|  | ADH4 | -1.852 | 1.533E-03 | 2.344E-02 |
|  | TRH | -1.842 | 6.954E-04 | 1.063E-02 |
|  | COLEC10 | -1.794 | 1.683E-04 | 2.573E-03 |
|  | PSG4 | -1.776 | 9.009E-04 | 1.377E-02 |
|  | HCN1 | -1.759 | 1.441E-03 | 2.203E-02 |
|  | HTR3A | -1.733 | 2.487E-03 | 3.802E-02 |
|  | FCRL5 | -1.726 | 1.327E-03 | 2.029E-02 |
|  | PIGR | -1.710 | 1.954E-03 | 2.988E-02 |
|  | LIPH | -1.683 | 3.596E-04 | 5.497E-03 |
|  | TNFRSF13B | -1.668 | 8.396E-04 | 1.284E-02 |
|  | NRG3 | -1.652 | 2.641E-03 | 4.037E-02 |
|  | SLC1A6 | -1.629 | 2.264E-03 | 3.461E-02 |
|  | CSF3 | -1.600 | 3.072E-03 | 4.697E-02 |
|  | C4BPB | -1.596 | 6.616E-05 | 1.011E-03 |
|  | FBP2 | -1.565 | 5.470E-04 | 8.362E-03 |
|  | PKHD1L1 | -1.556 | 7.205E-04 | 1.101E-02 |
|  | CASR | -1.556 | 2.987E-03 | 4.566E-02 |
|  | MYH13 | -1.552 | 7.021E-05 | 1.073E-03 |
|  | ASCL1 | -1.548 | 1.966E-04 | 3.005E-03 |
|  | C4BPA | -1.526 | 2.672E-04 | 4.085E-03 |
|  | PAH | -1.524 | 6.080E-04 | 9.296E-03 |
|  | EPHA10 | -1.495 | 1.591E-04 | 2.432E-03 |
|  | SLC6A13 | -1.466 | 1.756E-03 | 2.684E-02 |
|  | HS3ST4 | -1.463 | 2.415E-03 | 3.693E-02 |
|  | CD19 | -1.461 | 1.559E-04 | 2.384E-03 |
|  | MS4A1 | -1.454 | 1.104E-03 | 1.687E-02 |
|  | CAPN9 | -1.440 | 2.560E-03 | 3.913E-02 |
|  | LPA | -1.424 | 4.114E-04 | 6.290E-03 |
|  | FOXJ1 | -1.399 | 6.004E-04 | 9.179E-03 |
|  | MGAT4C | -1.393 | 1.669E-03 | 2.551E-02 |
|  | HP | -1.385 | 1.765E-03 | 2.698E-02 |
|  | TCF21 | -1.385 | 1.880E-05 | 2.874E-04 |
|  | CREB3L3 | -1.383 | 2.566E-03 | 3.923E-02 |
|  | RAB25 | -1.355 | 2.237E-03 | 3.419E-02 |
|  | CST2 | -1.342 | 7.072E-04 | 1.081E-02 |
|  | ACOT12 | -1.339 | 7.597E-04 | 1.161E-02 |
|  | C9 | -1.336 | 5.835E-07 | 8.920E-06 |
|  | CNGB1 | -1.304 | 1.938E-03 | 2.962E-02 |
|  | GDF6 | -1.299 | 3.585E-06 | 5.480E-05 |
|  | SLC7A4 | -1.295 | 9.447E-05 | 1.444E-03 |
|  | SCN7A | -1.289 | 1.150E-03 | 1.758E-02 |
|  | TPH1 | -1.289 | 9.368E-04 | 1.432E-02 |
|  | DNAH9 | -1.278 | 7.355E-05 | 1.124E-03 |
|  | PCK1 | -1.274 | 2.891E-03 | 4.420E-02 |
|  | C1orf94 | -1.268 | 1.081E-03 | 1.653E-02 |
|  | CHL1 | -1.241 | 1.333E-04 | 2.037E-03 |
|  | STK31 | -1.240 | 1.756E-03 | 2.684E-02 |
|  | SCTR | -1.238 | 9.770E-04 | 1.494E-02 |
|  | FCER2 | -1.236 | 2.733E-04 | 4.178E-03 |
|  | PAGE2B | -1.234 | 3.223E-03 | 4.927E-02 |
|  | TCERG1L | -1.222 | 2.924E-03 | 4.470E-02 |
|  | TSKS | -1.206 | 3.741E-05 | 5.719E-04 |
|  | LCN2 | -1.190 | 1.770E-03 | 2.705E-02 |
|  | GRIP2 | -1.186 | 2.176E-05 | 3.327E-04 |
|  | BFSP2 | -1.179 | 3.779E-04 | 5.778E-03 |
|  | ADH1A | -1.170 | 1.474E-03 | 2.253E-02 |
|  | CCR3 | -1.147 | 8.499E-04 | 1.299E-02 |
|  | HPSE2 | -1.139 | 2.256E-03 | 3.449E-02 |
|  | MMP27 | -1.129 | 2.605E-03 | 3.983E-02 |
|  | DSC1 | -1.129 | 2.663E-03 | 4.070E-02 |
|  | NXF3 | -1.126 | 3.421E-04 | 5.230E-03 |
|  | CLDN16 | -1.122 | 3.196E-03 | 4.885E-02 |
|  | GDPD4 | -1.114 | 5.621E-05 | 8.593E-04 |
|  | ST6GALNAC1 | -1.111 | 2.895E-03 | 4.426E-02 |
|  | POU1F1 | -1.110 | 2.249E-03 | 3.439E-02 |
|  | NPFFR1 | -1.110 | 1.310E-03 | 2.003E-02 |
|  | SYT16 | -1.105 | 1.153E-03 | 1.763E-02 |
|  | CNTN2 | -1.104 | 2.660E-03 | 4.066E-02 |
|  | SHANK2 | -1.101 | 3.084E-03 | 4.714E-02 |
|  | PTPRZ1 | -1.098 | 1.327E-03 | 2.028E-02 |
|  | COL4A3 | -1.092 | 3.521E-04 | 5.383E-03 |
|  | PTPN5 | -1.090 | 2.348E-03 | 3.590E-02 |
|  | TLR10 | -1.078 | 2.688E-04 | 4.109E-03 |
|  | BCAS1 | -1.067 | 2.990E-04 | 4.570E-03 |
|  | GPR55 | -1.065 | 8.098E-05 | 1.238E-03 |
|  | POLR2F | -1.056 | 1.865E-04 | 2.852E-03 |
|  | OR2L13 | -1.054 | 2.289E-03 | 3.499E-02 |
|  | TNFRSF17 | -1.045 | 2.656E-03 | 4.060E-02 |
|  | ADH1B | -1.036 | 2.404E-04 | 3.675E-03 |
|  | MLC1 | -1.035 | 1.502E-03 | 2.296E-02 |
|  | CLEC1B | -1.034 | 1.598E-04 | 2.443E-03 |
|  | TCF23 | -1.023 | 2.055E-03 | 3.141E-02 |
|  | IGFBP1 | -1.012 | 4.013E-04 | 6.135E-03 |
|  | CCL11 | -0.998 | 2.783E-04 | 4.255E-03 |
|  | SERPINA5 | -0.997 | 4.038E-04 | 6.173E-03 |
|  | IL4 | -0.993 | 1.079E-05 | 1.650E-04 |
|  | NAT8 | -0.993 | 1.255E-03 | 1.919E-02 |
|  | APOB | -0.990 | 2.971E-03 | 4.542E-02 |
|  | IRX1 | -0.987 | 1.469E-03 | 2.245E-02 |
|  | MPO | -0.980 | 2.809E-04 | 4.294E-03 |
|  | PTPRN | -0.962 | 1.630E-03 | 2.492E-02 |
|  | GCKR | -0.961 | 4.884E-04 | 7.467E-03 |
|  | LIPC | -0.958 | 9.477E-05 | 1.449E-03 |
|  | TTLL9 | -0.957 | 1.831E-04 | 2.799E-03 |
|  | ZNF541 | -0.946 | 2.721E-05 | 4.159E-04 |
|  | AKAP14 | -0.946 | 1.529E-03 | 2.338E-02 |
|  | SCARA5 | -0.945 | 4.187E-05 | 6.401E-04 |
|  | VIT | -0.943 | 7.375E-05 | 1.128E-03 |
|  | ABCA6 | -0.942 | 7.860E-05 | 1.202E-03 |
|  | LRAT | -0.936 | 3.270E-03 | 4.998E-02 |
|  | ROPN1L | -0.930 | 4.206E-06 | 6.430E-05 |
|  | DNAH5 | -0.920 | 1.805E-03 | 2.759E-02 |
|  | ABCG5 | -0.913 | 1.457E-03 | 2.227E-02 |
|  | DKK2 | -0.911 | 1.256E-05 | 1.920E-04 |
|  | PI16 | -0.909 | 4.817E-04 | 7.364E-03 |
|  | DHH | -0.907 | 1.630E-03 | 2.493E-02 |
|  | POU2AF1 | -0.907 | 1.654E-03 | 2.529E-02 |
|  | WNT4 | -0.905 | 2.886E-03 | 4.412E-02 |
|  | SLC1A7 | -0.901 | 1.109E-04 | 1.695E-03 |
|  | KRTAP5-10 | -0.900 | 1.120E-03 | 1.713E-02 |
|  | OR2W3 | -0.899 | 2.826E-03 | 4.320E-02 |
|  | ABCA9 | -0.894 | 2.163E-04 | 3.307E-03 |
|  | PLA2G2A | -0.885 | 1.787E-04 | 2.732E-03 |
|  | IGF1 | -0.883 | 1.234E-05 | 1.887E-04 |
|  | CCL20 | -0.881 | 6.554E-04 | 1.002E-02 |
|  | LAD1 | -0.881 | 2.809E-04 | 4.294E-03 |
|  | LTF | -0.880 | 2.036E-04 | 3.113E-03 |
|  | CALCR | -0.869 | 3.950E-04 | 6.038E-03 |
|  | IL1RAPL1 | -0.866 | 1.273E-03 | 1.945E-02 |
|  | WNT10A | -0.864 | 1.191E-03 | 1.820E-02 |
|  | DEFB1 | -0.863 | 1.543E-03 | 2.359E-02 |
|  | C10orf67 | -0.862 | 1.987E-03 | 3.038E-02 |
|  | WNT1 | -0.860 | 2.003E-03 | 3.062E-02 |
|  | COL4A4 | -0.859 | 1.195E-03 | 1.827E-02 |
|  | ZNF536 | -0.839 | 1.811E-03 | 2.769E-02 |
|  | SIGLEC8 | -0.836 | 4.629E-04 | 7.076E-03 |
|  | DIRC1 | -0.836 | 1.967E-03 | 3.008E-02 |
|  | ACSL6 | -0.835 | 2.051E-03 | 3.136E-02 |
|  | IRX2 | -0.830 | 2.044E-03 | 3.124E-02 |
|  | ABCB1 | -0.823 | 5.554E-06 | 8.490E-05 |
|  | S100A14 | -0.816 | 1.762E-04 | 2.693E-03 |
|  | GABRP | -0.815 | 2.067E-03 | 3.159E-02 |
|  | SLC4A9 | -0.813 | 1.364E-03 | 2.085E-02 |
|  | SGPP2 | -0.811 | 8.058E-05 | 1.232E-03 |
|  | CYP7A1 | -0.810 | 2.995E-03 | 4.578E-02 |
|  | CNNM1 | -0.809 | 2.133E-03 | 3.261E-02 |
|  | NDP | -0.808 | 1.356E-04 | 2.072E-03 |
|  | KLHDC8A | -0.807 | 1.886E-03 | 2.884E-02 |
|  | CCDC42 | -0.796 | 9.750E-05 | 1.491E-03 |
|  | TF | -0.782 | 9.593E-04 | 1.467E-02 |
|  | PLP1 | -0.781 | 1.937E-03 | 2.962E-02 |
|  | APOF | -0.774 | 1.428E-03 | 2.184E-02 |
|  | SAA1 | -0.772 | 2.582E-03 | 3.947E-02 |
|  | TFAP2A | -0.772 | 1.272E-03 | 1.944E-02 |
|  | GRHL1 | -0.771 | 2.691E-04 | 4.114E-03 |
|  | ADH1C | -0.769 | 2.677E-03 | 4.092E-02 |
|  | GHRL | -0.764 | 5.916E-04 | 9.045E-03 |
|  | CXCL6 | -0.760 | 2.180E-03 | 3.332E-02 |
|  | PLA2G5 | -0.759 | 3.012E-04 | 4.605E-03 |
|  | CLIC6 | -0.757 | 2.283E-04 | 3.490E-03 |
|  | SLC26A9 | -0.756 | 1.380E-03 | 2.110E-02 |
|  | THPO | -0.750 | 5.575E-05 | 8.524E-04 |
|  | ABCA10 | -0.749 | 1.665E-03 | 2.546E-02 |
|  | MIXL1 | -0.736 | 1.633E-03 | 2.497E-02 |
|  | MAL | -0.735 | 2.919E-03 | 4.462E-02 |
|  | CCL22 | -0.730 | 1.141E-03 | 1.745E-02 |
|  | LILRA2 | -0.729 | 9.399E-06 | 1.437E-04 |
|  | KIAA1257 | -0.729 | 1.062E-03 | 1.623E-02 |
|  | KCNH1 | -0.728 | 2.504E-03 | 3.828E-02 |
|  | CD1E | -0.727 | 2.041E-03 | 3.120E-02 |
|  | ZNF208 | -0.726 | 1.586E-03 | 2.425E-02 |
|  | WNT2 | -0.719 | 3.236E-03 | 4.948E-02 |
|  | NCR1 | -0.717 | 9.782E-04 | 1.495E-02 |
|  | KRT17 | -0.713 | 2.029E-03 | 3.102E-02 |
|  | KCNK15 | -0.711 | 8.466E-05 | 1.294E-03 |
|  | GRIK2 | -0.700 | 2.709E-03 | 4.142E-02 |
|  | IL12RB2 | -0.697 | 3.557E-04 | 5.438E-03 |
|  | TMPRSS13 | -0.694 | 2.745E-04 | 4.197E-03 |
|  | SLC39A12 | -0.690 | 1.922E-03 | 2.938E-02 |
|  | CCL17 | -0.687 | 8.179E-04 | 1.250E-02 |
|  | ITGB8 | -0.684 | 1.881E-03 | 2.875E-02 |
|  | CYP11A1 | -0.682 | 1.702E-03 | 2.602E-02 |
|  | KRT19 | -0.681 | 2.412E-03 | 3.687E-02 |
|  | ILDR1 | -0.681 | 2.603E-03 | 3.979E-02 |
|  | SYN3 | -0.680 | 1.606E-03 | 2.456E-02 |
|  | PCBP3 | -0.680 | 3.060E-04 | 4.678E-03 |
|  | CD207 | -0.680 | 1.676E-03 | 2.562E-02 |
|  | MFRP | -0.676 | 3.003E-04 | 4.590E-03 |
|  | C1orf167 | -0.675 | 1.866E-03 | 2.852E-02 |
|  | ELOVL3 | -0.671 | 1.185E-03 | 1.811E-02 |
|  | ABCA8 | -0.669 | 4.301E-04 | 6.575E-03 |
|  | MEP1B | -0.669 | 1.059E-03 | 1.619E-02 |
|  | DPEP3 | -0.667 | 1.564E-03 | 2.391E-02 |
|  | SLC16A4 | -0.662 | 2.814E-05 | 4.301E-04 |
|  | P2RY12 | -0.662 | 3.506E-04 | 5.360E-03 |
|  | ANXA3 | -0.657 | 6.670E-04 | 1.020E-02 |
|  | SH2D1B | -0.655 | 7.257E-04 | 1.109E-02 |
|  | TDRD10 | -0.655 | 1.802E-04 | 2.755E-03 |
|  | NFE2 | -0.654 | 1.444E-03 | 2.208E-02 |
|  | ASPA | -0.651 | 5.097E-04 | 7.793E-03 |
|  | STX19 | -0.651 | 1.431E-03 | 2.187E-02 |
|  | CEBPE | -0.648 | 5.472E-04 | 8.365E-03 |
|  | DMRT3 | -0.645 | 1.432E-03 | 2.189E-02 |
|  | CD79A | -0.642 | 1.967E-03 | 3.008E-02 |
|  | TEKT1 | -0.639 | 1.623E-03 | 2.480E-02 |
|  | GPR82 | -0.637 | 1.669E-03 | 2.552E-02 |
|  | DKK1 | -0.635 | 1.036E-03 | 1.584E-02 |
|  | CHRD | -0.632 | 3.872E-06 | 5.920E-05 |
|  | GPR18 | -0.631 | 2.128E-03 | 3.253E-02 |
|  | NGFR | -0.631 | 9.492E-05 | 1.451E-03 |
|  | CD38 | -0.631 | 6.686E-04 | 1.022E-02 |
|  | CLDN1 | -0.628 | 1.245E-03 | 1.903E-02 |
|  | ALDH1A3 | -0.627 | 1.621E-04 | 2.478E-03 |
|  | KCNG2 | -0.627 | 2.036E-03 | 3.113E-02 |
|  | SAMD12 | -0.626 | 2.830E-03 | 4.327E-02 |
|  | MED12L | -0.626 | 2.830E-04 | 4.326E-03 |
|  | F2RL2 | -0.626 | 2.108E-03 | 3.222E-02 |
|  | P2RY13 | -0.623 | 1.085E-04 | 1.659E-03 |
|  | CDH26 | -0.622 | 2.384E-03 | 3.645E-02 |
|  | TNF | -0.621 | 6.560E-04 | 1.003E-02 |
|  | WNT2B | -0.617 | 1.817E-03 | 2.778E-02 |
|  | LYPD3 | -0.616 | 6.447E-05 | 9.857E-04 |
|  | SGCG | -0.616 | 2.826E-03 | 4.321E-02 |
|  | HIPK4 | -0.615 | 1.711E-04 | 2.616E-03 |
|  | GPBAR1 | -0.614 | 5.357E-04 | 8.190E-03 |
|  | ZDHHC19 | -0.613 | 2.145E-03 | 3.279E-02 |
|  | PRKCZ | -0.613 | 2.230E-03 | 3.409E-02 |
|  | PADI4 | -0.611 | 2.332E-03 | 3.565E-02 |
|  | KCNJ1 | -0.610 | 1.124E-03 | 1.719E-02 |
|  | FUT2 | -0.604 | 2.280E-03 | 3.486E-02 |
|  | TIMD4 | -0.601 | 3.125E-03 | 4.778E-02 |
|  | ZBP1 | -0.599 | 2.804E-03 | 4.286E-02 |
|  | ASGR2 | -0.598 | 1.279E-04 | 1.955E-03 |
|  | FAM43B | -0.596 | 1.280E-03 | 1.957E-02 |
|  | IRF4 | -0.591 | 1.490E-03 | 2.278E-02 |
|  | PLEKHB1 | -0.591 | 3.100E-03 | 4.740E-02 |
|  | LRRC43 | -0.590 | 1.402E-03 | 2.143E-02 |
|  | HSH2D | -0.590 | 2.434E-03 | 3.721E-02 |
|  | TIMP3 | -0.589 | 3.068E-06 | 4.690E-05 |
|  | PLAC8 | -0.586 | 4.250E-04 | 6.497E-03 |
|  | TEKT3 | -0.585 | 9.273E-04 | 1.418E-02 |
|  | SLC28A1 | -0.582 | 2.373E-03 | 3.627E-02 |
|  | RIPK3 | -0.581 | 5.305E-06 | 8.110E-05 |
|  | DOK6 | -0.581 | 1.344E-03 | 2.055E-02 |
|  | AQP3 | -0.580 | 8.657E-05 | 1.324E-03 |
|  | CEACAM4 | -0.576 | 3.118E-04 | 4.767E-03 |
|  | TNXB | -0.573 | 7.749E-04 | 1.185E-02 |
|  | AVPR2 | -0.571 | 4.115E-04 | 6.290E-03 |
|  | CMYA5 | -0.570 | 2.215E-03 | 3.386E-02 |
|  | DTX1 | -0.565 | 1.799E-03 | 2.750E-02 |
|  | LTC4S | -0.560 | 2.012E-03 | 3.076E-02 |
|  | GFRA1 | -0.555 | 8.536E-04 | 1.305E-02 |
|  | FNDC5 | -0.555 | 6.913E-05 | 1.057E-03 |
|  | LIF | -0.553 | 9.559E-05 | 1.461E-03 |
|  | BMP8A | -0.550 | 1.582E-04 | 2.419E-03 |
|  | PLEKHA6 | -0.546 | 2.287E-03 | 3.496E-02 |
|  | DNAH17 | -0.546 | 2.778E-03 | 4.247E-02 |
|  | SLC24A4 | -0.545 | 2.405E-03 | 3.677E-02 |
|  | SLC16A8 | -0.543 | 2.000E-03 | 3.057E-02 |
|  | S100B | -0.542 | 2.808E-03 | 4.293E-02 |
|  | MT1A | -0.539 | 9.524E-04 | 1.456E-02 |
|  | AKR1C2 | -0.539 | 1.671E-03 | 2.554E-02 |
|  | INSL3 | -0.538 | 3.064E-03 | 4.684E-02 |
|  | S100Z | -0.536 | 9.387E-04 | 1.435E-02 |
|  | LRG1 | -0.532 | 1.415E-03 | 2.164E-02 |
|  | ZBTB32 | -0.527 | 1.856E-03 | 2.837E-02 |
|  | EFHD1 | -0.526 | 2.515E-05 | 3.844E-04 |
|  | XPNPEP2 | -0.525 | 1.608E-03 | 2.458E-02 |
|  | TNFRSF8 | -0.524 | 1.045E-03 | 1.597E-02 |
|  | BAMBI | -0.523 | 1.000E-04 | 1.529E-03 |
|  | TGFA | -0.515 | 6.550E-04 | 1.001E-02 |
|  | CD1C | -0.513 | 2.438E-03 | 3.727E-02 |
|  | SIGLEC11 | -0.511 | 2.506E-03 | 3.831E-02 |
|  | HAL | -0.511 | 1.338E-03 | 2.046E-02 |
|  | ABAT | -0.508 | 3.081E-05 | 4.710E-04 |
|  | DISC1 | -0.508 | 2.590E-06 | 3.960E-05 |
|  | ZNF423 | -0.508 | 5.826E-04 | 8.906E-03 |
|  | MT1F | -0.507 | 1.808E-04 | 2.763E-03 |
|  | SERPINF2 | -0.507 | 3.975E-05 | 6.077E-04 |
|  | REM1 | -0.505 | 3.474E-04 | 5.312E-03 |
|  | FCER1A | -0.503 | 1.918E-03 | 2.933E-02 |
|  | CR1 | -0.503 | 2.919E-03 | 4.463E-02 |
|  | ICAM3 | -0.502 | 3.034E-03 | 4.638E-02 |
|  | SLC13A3 | -0.500 | 2.826E-04 | 4.320E-03 |

*FC* Fold change; *FDR* False Discovery Rate

**Table S2 The list of differentially expressed genes between high and low risk groups in training dataset.**

| **Regulation** | **Gene Symbol** | **Log_2_FC** | **P value** | **FDR** |
| --- | --- | --- | --- | --- |
| *Up-regulation* | HTR2A | 0.503 | 2.488E-03 | 2.016E-02 |
|  | CSRP2 | 0.504 | 1.856E-05 | 1.503E-04 |
|  | SGIP1 | 0.505 | 4.320E-03 | 3.499E-02 |
|  | SNAP25 | 0.507 | 1.164E-03 | 9.430E-03 |
|  | HIST1H3C | 0.509 | 4.688E-03 | 3.797E-02 |
|  | EYA4 | 0.510 | 2.351E-03 | 1.905E-02 |
|  | GJB2 | 0.513 | 5.263E-03 | 4.263E-02 |
|  | SLC16A10 | 0.515 | 1.848E-03 | 1.497E-02 |
|  | NTNG1 | 0.520 | 3.403E-03 | 2.757E-02 |
|  | CHRNA5 | 0.521 | 2.842E-04 | 2.302E-03 |
|  | PPARGC1A | 0.523 | 4.378E-03 | 3.546E-02 |
|  | HIST1H2AD | 0.528 | 1.530E-03 | 1.239E-02 |
|  | SPOCD1 | 0.532 | 1.819E-03 | 1.473E-02 |
|  | PHEX | 0.533 | 3.105E-03 | 2.515E-02 |
|  | TMEM108 | 0.534 | 1.972E-04 | 1.598E-03 |
|  | GREM1 | 0.537 | 3.850E-03 | 3.119E-02 |
|  | CH25H | 0.537 | 3.723E-05 | 3.015E-04 |
|  | CHST6 | 0.540 | 2.277E-03 | 1.844E-02 |
|  | PI15 | 0.540 | 2.479E-03 | 2.008E-02 |
|  | DTNA | 0.542 | 7.015E-04 | 5.682E-03 |
|  | CPLX4 | 0.546 | 4.815E-03 | 3.900E-02 |
|  | CA12 | 0.547 | 1.037E-05 | 8.400E-05 |
|  | SMOC1 | 0.556 | 9.707E-04 | 7.862E-03 |
|  | CPEB1 | 0.560 | 7.389E-05 | 5.986E-04 |
|  | TRIM9 | 0.580 | 1.238E-04 | 1.003E-03 |
|  | RBM24 | 0.583 | 2.179E-03 | 1.765E-02 |
|  | NEK10 | 0.583 | 4.857E-03 | 3.934E-02 |
|  | CHI3L2 | 0.585 | 1.295E-03 | 1.049E-02 |
|  | CHRDL2 | 0.588 | 3.741E-03 | 3.030E-02 |
|  | ITGA8 | 0.590 | 9.620E-04 | 7.792E-03 |
|  | PPFIA2 | 0.590 | 1.468E-03 | 1.189E-02 |
|  | SUMO4 | 0.607 | 2.838E-04 | 2.299E-03 |
|  | LRRC15 | 0.613 | 3.148E-03 | 2.550E-02 |
|  | ARG2 | 0.618 | 2.768E-05 | 2.242E-04 |
|  | TMEM74 | 0.629 | 3.105E-03 | 2.515E-02 |
|  | EFHC2 | 0.630 | 6.700E-04 | 5.427E-03 |
|  | ADAM21 | 0.631 | 1.196E-03 | 9.688E-03 |
|  | GRIN2A | 0.633 | 6.089E-03 | 4.932E-02 |
|  | GLIS3 | 0.650 | 1.556E-06 | 1.260E-05 |
|  | GDF5 | 0.653 | 1.451E-03 | 1.175E-02 |
|  | HOXB9 | 0.662 | 1.115E-03 | 9.028E-03 |
|  | MAP3K15 | 0.664 | 4.784E-03 | 3.875E-02 |
|  | ELAVL2 | 0.665 | 5.744E-03 | 4.652E-02 |
|  | BMPR1B | 0.669 | 3.087E-04 | 2.501E-03 |
|  | ST6GAL2 | 0.674 | 1.456E-04 | 1.179E-03 |
|  | XK | 0.685 | 4.905E-03 | 3.973E-02 |
|  | CPA4 | 0.691 | 5.037E-03 | 4.080E-02 |
|  | CNTN1 | 0.708 | 2.383E-03 | 1.930E-02 |
|  | DNAH11 | 0.722 | 2.868E-03 | 2.323E-02 |
|  | RASL10B | 0.725 | 7.358E-06 | 5.960E-05 |
|  | OTX1 | 0.727 | 1.673E-03 | 1.355E-02 |
|  | MAPT | 0.727 | 7.414E-04 | 6.006E-03 |
|  | IGSF10 | 0.734 | 1.462E-03 | 1.184E-02 |
|  | MLPH | 0.735 | 5.890E-04 | 4.771E-03 |
|  | HS3ST2 | 0.746 | 4.035E-04 | 3.268E-03 |
|  | HIST1H1D | 0.746 | 8.135E-04 | 6.590E-03 |
|  | LRFN5 | 0.749 | 1.192E-03 | 9.656E-03 |
|  | PITX2 | 0.751 | 3.082E-04 | 2.497E-03 |
|  | BBOX1 | 0.768 | 9.699E-04 | 7.856E-03 |
|  | LRRC8E | 0.776 | 1.295E-03 | 1.049E-02 |
|  | HIST1H2BI | 0.785 | 2.715E-03 | 2.199E-02 |
|  | ZMAT4 | 0.794 | 4.291E-03 | 3.475E-02 |
|  | FGF5 | 0.801 | 4.769E-03 | 3.863E-02 |
|  | RPA4 | 0.803 | 1.091E-04 | 8.838E-04 |
|  | SLC2A12 | 0.827 | 2.147E-04 | 1.739E-03 |
|  | HTR7 | 0.841 | 3.810E-04 | 3.086E-03 |
|  | WNT7B | 0.844 | 4.915E-03 | 3.981E-02 |
|  | ZP1 | 0.850 | 1.070E-03 | 8.664E-03 |
|  | PRKG2 | 0.864 | 4.045E-03 | 3.277E-02 |
|  | CA9 | 0.875 | 1.083E-05 | 8.770E-05 |
|  | CDH18 | 0.879 | 5.508E-03 | 4.461E-02 |
|  | SBSN | 0.890 | 4.708E-03 | 3.813E-02 |
|  | MMP10 | 0.891 | 2.680E-03 | 2.171E-02 |
|  | KY | 0.899 | 4.400E-03 | 3.564E-02 |
|  | ALDH1L1 | 0.903 | 1.577E-03 | 1.278E-02 |
|  | CKMT2 | 0.903 | 1.086E-04 | 8.797E-04 |
|  | GPR143 | 0.921 | 4.012E-03 | 3.250E-02 |
|  | WFIKKN2 | 0.926 | 1.901E-03 | 1.540E-02 |
|  | IBSP | 0.928 | 5.292E-03 | 4.286E-02 |
|  | KCTD16 | 0.928 | 1.683E-03 | 1.363E-02 |
|  | MYH15 | 0.962 | 2.102E-03 | 1.703E-02 |
|  | OGDHL | 0.967 | 2.190E-03 | 1.774E-02 |
|  | RAB3B | 0.987 | 6.451E-04 | 5.225E-03 |
|  | PTPRN | 0.989 | 4.259E-03 | 3.450E-02 |
|  | LANCL3 | 0.991 | 1.666E-05 | 1.349E-04 |
|  | VSNL1 | 1.040 | 4.437E-05 | 3.594E-04 |
|  | OMG | 1.091 | 2.910E-03 | 2.357E-02 |
|  | ABCA13 | 1.117 | 3.915E-03 | 3.171E-02 |
|  | KCNJ15 | 1.132 | 1.395E-06 | 1.130E-05 |
|  | AKR1D1 | 1.160 | 5.036E-03 | 4.079E-02 |
|  | GADL1 | 1.174 | 3.466E-03 | 2.807E-02 |
|  | SLC22A2 | 1.199 | 1.138E-03 | 9.221E-03 |
|  | CPA6 | 1.225 | 1.507E-03 | 1.220E-02 |
|  | TRIM63 | 1.227 | 9.347E-04 | 7.571E-03 |
|  | GRM1 | 1.228 | 8.264E-04 | 6.694E-03 |
|  | TYRP1 | 1.235 | 5.735E-03 | 4.645E-02 |
|  | MOG | 1.250 | 2.785E-03 | 2.256E-02 |
|  | PRL | 1.252 | 3.259E-03 | 2.640E-02 |
|  | LHX8 | 1.305 | 1.140E-03 | 9.238E-03 |
|  | CHRNA9 | 1.323 | 2.104E-03 | 1.704E-02 |
|  | SULT1C2 | 1.327 | 1.478E-03 | 1.197E-02 |
|  | TTLL2 | 1.335 | 2.376E-03 | 1.925E-02 |
|  | ABCB5 | 1.348 | 1.021E-04 | 8.267E-04 |
|  | MMP13 | 1.361 | 1.199E-04 | 9.716E-04 |
|  | OSTN | 1.421 | 3.338E-03 | 2.704E-02 |
|  | TRDN | 1.430 | 3.406E-04 | 2.759E-03 |
|  | CPN2 | 1.478 | 4.846E-04 | 3.926E-03 |
|  | HDGFL1 | 1.546 | 3.146E-03 | 2.548E-02 |
|  | CRYBA4 | 1.556 | 2.003E-03 | 1.623E-02 |
|  | KLK4 | 1.649 | 3.517E-04 | 2.849E-03 |
|  | CCK | 1.759 | 7.777E-04 | 6.299E-03 |
|  | SYTL5 | 1.780 | 2.110E-05 | 1.709E-04 |
|  | NR1H4 | 1.850 | 3.781E-04 | 3.063E-03 |
|  | SLITRK1 | 1.967 | 2.962E-03 | 2.400E-02 |
|  | NR0B1 | 2.003 | 6.356E-04 | 5.149E-03 |
|  | GLRA3 | 2.219 | 1.146E-03 | 9.285E-03 |
|  | PNLIPRP3 | 2.270 | 1.189E-03 | 9.633E-03 |
| *Down-regulation* | MUC6 | -2.737 | 3.588E-03 | 2.907E-02 |
|  | CHRM1 | -2.618 | 4.968E-04 | 4.024E-03 |
|  | SLC35D3 | -2.216 | 5.679E-03 | 4.600E-02 |
|  | HS3ST4 | -2.200 | 2.900E-04 | 2.349E-03 |
|  | TDRD9 | -2.183 | 6.150E-05 | 4.981E-04 |
|  | EDAR | -2.155 | 1.371E-03 | 1.111E-02 |
|  | GRIN1 | -2.033 | 4.234E-03 | 3.429E-02 |
|  | CHRNA6 | -1.981 | 2.367E-03 | 1.918E-02 |
|  | CHST8 | -1.946 | 9.617E-04 | 7.789E-03 |
|  | CHRNA4 | -1.919 | 2.744E-03 | 2.223E-02 |
|  | GABRG1 | -1.846 | 4.110E-03 | 3.329E-02 |
|  | GRM4 | -1.828 | 2.830E-03 | 2.293E-02 |
|  | DSCAML1 | -1.816 | 6.662E-05 | 5.396E-04 |
|  | HES5 | -1.790 | 4.914E-06 | 3.980E-05 |
|  | NEUROD2 | -1.744 | 6.126E-03 | 4.962E-02 |
|  | NEU4 | -1.723 | 1.402E-03 | 1.135E-02 |
|  | C1orf94 | -1.719 | 4.024E-04 | 3.259E-03 |
|  | SPTA1 | -1.709 | 7.746E-05 | 6.274E-04 |
|  | LIPH | -1.705 | 1.410E-03 | 1.142E-02 |
|  | KCNT1 | -1.703 | 4.762E-03 | 3.857E-02 |
|  | FGF9 | -1.701 | 5.578E-05 | 4.519E-04 |
|  | BARX2 | -1.691 | 2.839E-03 | 2.299E-02 |
|  | GBX2 | -1.691 | 3.996E-04 | 3.237E-03 |
|  | KCNJ10 | -1.687 | 2.038E-04 | 1.651E-03 |
|  | ALOX15 | -1.662 | 4.733E-03 | 3.834E-02 |
|  | TPH1 | -1.659 | 1.017E-03 | 8.240E-03 |
|  | CAPN9 | -1.643 | 5.249E-03 | 4.252E-02 |
|  | SHANK2 | -1.626 | 6.372E-04 | 5.161E-03 |
|  | CCDC33 | -1.605 | 2.635E-03 | 2.134E-02 |
|  | MS4A1 | -1.584 | 2.494E-03 | 2.020E-02 |
|  | TDGF1 | -1.530 | 4.381E-03 | 3.549E-02 |
|  | LEP | -1.518 | 3.695E-03 | 2.993E-02 |
|  | WNT4 | -1.516 | 6.763E-05 | 5.478E-04 |
|  | FCRL3 | -1.507 | 1.133E-04 | 9.178E-04 |
|  | TNFRSF13B | -1.500 | 4.986E-03 | 4.038E-02 |
|  | CD19 | -1.487 | 5.511E-04 | 4.464E-03 |
|  | AJAP1 | -1.471 | 6.237E-04 | 5.052E-03 |
|  | TEKT4 | -1.451 | 3.988E-04 | 3.230E-03 |
|  | CRHR1 | -1.441 | 3.380E-03 | 2.738E-02 |
|  | OLIG1 | -1.434 | 6.139E-03 | 4.973E-02 |
|  | CST2 | -1.420 | 6.602E-04 | 5.348E-03 |
|  | DACH2 | -1.390 | 5.412E-03 | 4.384E-02 |
|  | SLC24A4 | -1.371 | 6.444E-07 | 5.220E-06 |
|  | LRRC38 | -1.367 | 2.335E-03 | 1.892E-02 |
|  | TMPRSS3 | -1.366 | 2.014E-03 | 1.631E-02 |
|  | GPR55 | -1.348 | 4.256E-05 | 3.447E-04 |
|  | ST6GALNAC1 | -1.337 | 5.186E-03 | 4.200E-02 |
|  | TNFRSF17 | -1.336 | 2.075E-03 | 1.681E-02 |
|  | WNT2 | -1.335 | 2.570E-05 | 2.082E-04 |
|  | MEGF11 | -1.329 | 2.291E-03 | 1.856E-02 |
|  | PRSS8 | -1.322 | 6.021E-03 | 4.877E-02 |
|  | CXXC4 | -1.321 | 2.531E-04 | 2.050E-03 |
|  | DACT2 | -1.319 | 3.458E-04 | 2.801E-03 |
|  | CNTN2 | -1.313 | 3.177E-03 | 2.573E-02 |
|  | GPR31 | -1.311 | 4.916E-04 | 3.982E-03 |
|  | MAFA | -1.285 | 5.695E-03 | 4.613E-02 |
|  | GRIN2C | -1.285 | 1.388E-04 | 1.124E-03 |
|  | DBH | -1.281 | 8.868E-05 | 7.183E-04 |
|  | WNT1 | -1.264 | 3.309E-04 | 2.680E-03 |
|  | PTPN5 | -1.255 | 4.805E-03 | 3.892E-02 |
|  | KCNJ9 | -1.248 | 5.570E-04 | 4.512E-03 |
|  | CD1B | -1.241 | 8.695E-04 | 7.043E-03 |
|  | C22orf15 | -1.235 | 1.342E-03 | 1.087E-02 |
|  | HAO2 | -1.233 | 1.246E-03 | 1.010E-02 |
|  | SCN7A | -1.224 | 3.682E-03 | 2.982E-02 |
|  | SPIC | -1.223 | 3.404E-03 | 2.757E-02 |
|  | ENTPD8 | -1.223 | 5.533E-03 | 4.482E-02 |
|  | MPPED1 | -1.223 | 4.007E-03 | 3.245E-02 |
|  | ERBB3 | -1.213 | 2.123E-03 | 1.719E-02 |
|  | C4BPB | -1.210 | 1.581E-03 | 1.281E-02 |
|  | KIR3DL2 | -1.207 | 1.664E-03 | 1.348E-02 |
|  | BCL2L14 | -1.207 | 5.044E-04 | 4.086E-03 |
|  | SLC12A3 | -1.205 | 6.462E-04 | 5.234E-03 |
|  | CCR9 | -1.201 | 5.403E-03 | 4.376E-02 |
|  | CLEC9A | -1.193 | 7.130E-05 | 5.776E-04 |
|  | BTNL2 | -1.192 | 4.284E-03 | 3.470E-02 |
|  | ABCA10 | -1.192 | 2.336E-05 | 1.892E-04 |
|  | TTC16 | -1.190 | 4.469E-06 | 3.620E-05 |
|  | NPFFR1 | -1.189 | 3.345E-03 | 2.709E-02 |
|  | KIF19 | -1.186 | 1.326E-05 | 1.074E-04 |
|  | EPHA10 | -1.183 | 3.213E-03 | 2.602E-02 |
|  | FCER2 | -1.180 | 1.444E-03 | 1.170E-02 |
|  | FAM19A2 | -1.178 | 8.916E-04 | 7.222E-03 |
|  | B3GAT1 | -1.177 | 1.724E-04 | 1.396E-03 |
|  | IL12B | -1.177 | 3.793E-04 | 3.072E-03 |
|  | ZNF80 | -1.171 | 2.209E-03 | 1.789E-02 |
|  | COL9A1 | -1.171 | 2.895E-03 | 2.345E-02 |
|  | PRSS21 | -1.170 | 2.605E-03 | 2.110E-02 |
|  | RGS6 | -1.165 | 3.695E-03 | 2.993E-02 |
|  | MMP27 | -1.165 | 3.659E-03 | 2.964E-02 |
|  | MCF2 | -1.151 | 3.293E-03 | 2.667E-02 |
|  | SCARA5 | -1.150 | 8.580E-07 | 6.950E-06 |
|  | MOV10L1 | -1.147 | 6.592E-04 | 5.340E-03 |
|  | TREML2 | -1.143 | 3.344E-03 | 2.709E-02 |
|  | EVPL | -1.136 | 4.017E-03 | 3.254E-02 |
|  | PLA2G2D | -1.133 | 5.617E-04 | 4.550E-03 |
|  | P2RX5 | -1.131 | 1.952E-04 | 1.581E-03 |
|  | HPSE2 | -1.116 | 5.811E-03 | 4.707E-02 |
|  | MYCN | -1.115 | 2.954E-04 | 2.393E-03 |
|  | MAPK15 | -1.109 | 3.838E-03 | 3.109E-02 |
|  | FCN2 | -1.096 | 1.404E-03 | 1.137E-02 |
|  | FAM3B | -1.090 | 2.589E-03 | 2.097E-02 |
|  | CHL1 | -1.084 | 6.320E-04 | 5.119E-03 |
|  | KIAA1257 | -1.083 | 9.638E-05 | 7.807E-04 |
|  | SPDEF | -1.082 | 3.121E-03 | 2.528E-02 |
|  | SLC35F3 | -1.078 | 9.555E-04 | 7.740E-03 |
|  | RPRML | -1.076 | 2.215E-03 | 1.794E-02 |
|  | TMPRSS13 | -1.072 | 2.605E-06 | 2.110E-05 |
|  | SLIT1 | -1.071 | 1.539E-03 | 1.247E-02 |
|  | TLR10 | -1.069 | 1.147E-03 | 9.291E-03 |
|  | TPO | -1.067 | 2.262E-03 | 1.832E-02 |
|  | IRX2 | -1.060 | 4.940E-04 | 4.001E-03 |
|  | APOL5 | -1.056 | 1.613E-03 | 1.307E-02 |
|  | KIRREL3 | -1.055 | 7.365E-04 | 5.965E-03 |
|  | BTLA | -1.054 | 3.976E-04 | 3.221E-03 |
|  | GRIK3 | -1.053 | 3.762E-03 | 3.048E-02 |
|  | LILRA4 | -1.052 | 1.039E-03 | 8.418E-03 |
|  | SERPINA10 | -1.047 | 5.520E-03 | 4.471E-02 |
|  | UBASH3A | -1.042 | 3.683E-05 | 2.984E-04 |
|  | HMX3 | -1.036 | 7.185E-04 | 5.820E-03 |
|  | MAL | -1.029 | 6.002E-04 | 4.861E-03 |
|  | TNFSF14 | -1.020 | 2.901E-04 | 2.350E-03 |
|  | OR1L8 | -1.020 | 1.876E-05 | 1.519E-04 |
|  | LY9 | -1.019 | 4.430E-04 | 3.588E-03 |
|  | FASLG | -1.014 | 5.223E-05 | 4.231E-04 |
|  | SLC4A9 | -1.013 | 5.733E-04 | 4.644E-03 |
|  | LHX4 | -1.011 | 9.679E-04 | 7.840E-03 |
|  | AICDA | -1.009 | 2.882E-03 | 2.334E-02 |
|  | VWA2 | -1.004 | 1.761E-03 | 1.426E-02 |
|  | KIR2DL3 | -1.001 | 4.208E-03 | 3.409E-02 |
|  | POU2AF1 | -1.000 | 2.812E-03 | 2.278E-02 |
|  | LTA | -1.000 | 3.570E-05 | 2.892E-04 |
|  | CHAD | -0.999 | 6.937E-04 | 5.619E-03 |
|  | PI16 | -0.998 | 3.041E-04 | 2.463E-03 |
|  | GALR3 | -0.998 | 1.150E-03 | 9.311E-03 |
|  | GRB7 | -0.988 | 1.138E-03 | 9.219E-03 |
|  | DEGS2 | -0.973 | 1.181E-05 | 9.570E-05 |
|  | SDK2 | -0.972 | 9.503E-04 | 7.698E-03 |
|  | CD3G | -0.968 | 6.868E-05 | 5.563E-04 |
|  | CYP3A43 | -0.967 | 1.587E-03 | 1.286E-02 |
|  | FBXO47 | -0.967 | 4.409E-03 | 3.571E-02 |
|  | REM1 | -0.967 | 1.975E-08 | 1.600E-07 |
|  | DHH | -0.967 | 4.519E-03 | 3.660E-02 |
|  | OIT3 | -0.964 | 1.447E-04 | 1.172E-03 |
|  | SIGLEC8 | -0.958 | 4.516E-04 | 3.658E-03 |
|  | CLEC4C | -0.957 | 2.922E-03 | 2.367E-02 |
|  | TPTE2 | -0.956 | 9.450E-04 | 7.655E-03 |
|  | JAKMIP1 | -0.946 | 6.972E-05 | 5.647E-04 |
|  | GDPD4 | -0.945 | 9.774E-04 | 7.917E-03 |
|  | CD1E | -0.945 | 6.753E-04 | 5.470E-03 |
|  | TTYH1 | -0.943 | 2.928E-03 | 2.371E-02 |
|  | MATN4 | -0.938 | 4.416E-03 | 3.577E-02 |
|  | ANKK1 | -0.938 | 6.194E-04 | 5.017E-03 |
|  | OPN4 | -0.937 | 2.247E-03 | 1.820E-02 |
|  | KLK14 | -0.936 | 1.371E-03 | 1.111E-02 |
|  | PYHIN1 | -0.935 | 1.188E-04 | 9.623E-04 |
|  | LGR6 | -0.932 | 5.962E-03 | 4.830E-02 |
|  | SIGLEC11 | -0.930 | 3.494E-05 | 2.830E-04 |
|  | LMX1B | -0.925 | 2.193E-03 | 1.777E-02 |
|  | FZD10 | -0.924 | 2.359E-03 | 1.911E-02 |
|  | SGPP2 | -0.921 | 8.106E-05 | 6.566E-04 |
|  | NCR1 | -0.917 | 4.191E-04 | 3.395E-03 |
|  | EOMES | -0.914 | 6.105E-05 | 4.945E-04 |
|  | GIPR | -0.913 | 1.944E-04 | 1.574E-03 |
|  | GHRL | -0.912 | 9.124E-04 | 7.390E-03 |
|  | CD79A | -0.912 | 4.390E-04 | 3.556E-03 |
|  | RNASE13 | -0.910 | 5.738E-04 | 4.648E-03 |
|  | GABRP | -0.908 | 4.920E-03 | 3.985E-02 |
|  | KCNC3 | -0.905 | 2.559E-05 | 2.073E-04 |
|  | LAX1 | -0.903 | 7.060E-04 | 5.718E-03 |
|  | UROC1 | -0.899 | 4.069E-03 | 3.296E-02 |
|  | CD38 | -0.899 | 1.095E-05 | 8.870E-05 |
|  | ADRB1 | -0.892 | 2.997E-03 | 2.427E-02 |
|  | SCML4 | -0.888 | 1.345E-03 | 1.090E-02 |
|  | ADAMTSL3 | -0.887 | 7.392E-05 | 5.987E-04 |
|  | UPP2 | -0.887 | 1.154E-03 | 9.351E-03 |
|  | ECEL1 | -0.886 | 2.113E-03 | 1.712E-02 |
|  | TMEM52 | -0.885 | 2.636E-03 | 2.135E-02 |
|  | DKK2 | -0.882 | 6.810E-05 | 5.516E-04 |
|  | STX19 | -0.881 | 9.519E-04 | 7.710E-03 |
|  | BAIAP3 | -0.880 | 1.370E-03 | 1.109E-02 |
|  | IFNG | -0.879 | 1.895E-03 | 1.535E-02 |
|  | GPR18 | -0.879 | 6.013E-04 | 4.870E-03 |
|  | C9 | -0.877 | 6.242E-04 | 5.056E-03 |
|  | CYP2C8 | -0.876 | 5.429E-04 | 4.398E-03 |
|  | ZBP1 | -0.875 | 7.132E-04 | 5.777E-03 |
|  | NTNG2 | -0.869 | 2.948E-04 | 2.388E-03 |
|  | CRYBB3 | -0.867 | 4.786E-05 | 3.877E-04 |
|  | IZUMO1 | -0.864 | 5.741E-06 | 4.650E-05 |
|  | TMEM130 | -0.861 | 4.951E-03 | 4.010E-02 |
|  | LRRTM2 | -0.860 | 1.924E-03 | 1.559E-02 |
|  | GFRA1 | -0.858 | 5.506E-06 | 4.460E-05 |
|  | ICAM3 | -0.858 | 1.250E-04 | 1.012E-03 |
|  | KRTAP5-10 | -0.857 | 2.777E-03 | 2.249E-02 |
|  | GPR174 | -0.855 | 1.014E-03 | 8.211E-03 |
|  | ZAN | -0.851 | 3.734E-03 | 3.025E-02 |
|  | HSH2D | -0.848 | 5.718E-04 | 4.631E-03 |
|  | GPR82 | -0.848 | 4.112E-04 | 3.331E-03 |
|  | IL4 | -0.847 | 1.509E-04 | 1.222E-03 |
|  | LTC4S | -0.846 | 1.004E-04 | 8.133E-04 |
|  | ABCA6 | -0.845 | 3.162E-04 | 2.561E-03 |
|  | UBD | -0.845 | 6.482E-04 | 5.251E-03 |
|  | TRAT1 | -0.840 | 1.246E-03 | 1.009E-02 |
|  | USP6 | -0.839 | 1.440E-03 | 1.166E-02 |
|  | SEMA3E | -0.838 | 3.715E-03 | 3.009E-02 |
|  | ALDH1A3 | -0.838 | 1.037E-06 | 8.400E-06 |
|  | GRIP2 | -0.836 | 2.045E-03 | 1.656E-02 |
|  | CD1A | -0.836 | 5.230E-03 | 4.236E-02 |
|  | TIMD4 | -0.834 | 1.632E-03 | 1.322E-02 |
|  | BCAS1 | -0.833 | 2.919E-03 | 2.364E-02 |
|  | DNAH3 | -0.832 | 3.896E-03 | 3.155E-02 |
|  | GFRA2 | -0.831 | 4.688E-05 | 3.797E-04 |
|  | P2RY13 | -0.824 | 3.605E-06 | 2.920E-05 |
|  | COL4A4 | -0.823 | 3.817E-03 | 3.092E-02 |
|  | SH2D1A | -0.820 | 1.231E-04 | 9.973E-04 |
|  | SFTPD | -0.818 | 1.696E-03 | 1.374E-02 |
|  | NCR3 | -0.818 | 2.432E-04 | 1.970E-03 |
|  | ITGB7 | -0.817 | 1.304E-04 | 1.056E-03 |
|  | ZBED2 | -0.815 | 1.703E-03 | 1.380E-02 |
|  | ATP8A2 | -0.815 | 3.347E-03 | 2.711E-02 |
|  | FCRL6 | -0.813 | 1.356E-04 | 1.099E-03 |
|  | FUT7 | -0.812 | 4.001E-04 | 3.241E-03 |
|  | SLC28A3 | -0.809 | 5.715E-03 | 4.629E-02 |
|  | P2RY10 | -0.809 | 6.020E-04 | 4.876E-03 |
|  | CXCR3 | -0.807 | 4.112E-05 | 3.331E-04 |
|  | SELP | -0.805 | 8.158E-05 | 6.608E-04 |
|  | CAPN12 | -0.800 | 9.928E-04 | 8.042E-03 |
|  | TBX21 | -0.799 | 1.206E-04 | 9.770E-04 |
|  | WNT9B | -0.793 | 3.606E-03 | 2.921E-02 |
|  | CIB3 | -0.793 | 2.352E-03 | 1.905E-02 |
|  | NRG4 | -0.793 | 1.927E-03 | 1.561E-02 |
|  | CCL17 | -0.792 | 7.503E-04 | 6.078E-03 |
|  | CCL25 | -0.792 | 4.043E-03 | 3.275E-02 |
|  | SIRPD | -0.792 | 5.620E-03 | 4.552E-02 |
|  | ABCA9 | -0.792 | 8.548E-04 | 6.924E-03 |
|  | PRND | -0.790 | 1.659E-04 | 1.344E-03 |
|  | ITGAD | -0.789 | 1.643E-03 | 1.331E-02 |
|  | FGF18 | -0.788 | 8.839E-05 | 7.159E-04 |
|  | CYP11A1 | -0.786 | 9.413E-04 | 7.624E-03 |
|  | TNXB | -0.785 | 2.752E-05 | 2.229E-04 |
|  | SLAMF7 | -0.783 | 7.372E-05 | 5.972E-04 |
|  | CALCR | -0.783 | 2.785E-03 | 2.256E-02 |
|  | CRIP1 | -0.782 | 1.046E-07 | 8.470E-07 |
|  | MST1R | -0.778 | 1.563E-03 | 1.266E-02 |
|  | DEFB1 | -0.774 | 3.277E-03 | 2.654E-02 |
|  | GIMAP5 | -0.772 | 9.691E-06 | 7.850E-05 |
|  | LAT | -0.771 | 1.778E-05 | 1.440E-04 |
|  | PON3 | -0.770 | 5.872E-03 | 4.757E-02 |
|  | CCL22 | -0.763 | 2.247E-03 | 1.820E-02 |
|  | WNT2B | -0.761 | 4.938E-04 | 4.000E-03 |
|  | XCR1 | -0.760 | 2.293E-03 | 1.858E-02 |
|  | SULT1A2 | -0.758 | 8.444E-04 | 6.840E-03 |
|  | RIPK3 | -0.757 | 1.852E-08 | 1.500E-07 |
|  | TBC1D10C | -0.757 | 7.309E-06 | 5.920E-05 |
|  | DPEP3 | -0.757 | 1.899E-03 | 1.538E-02 |
|  | RAB37 | -0.756 | 1.775E-05 | 1.438E-04 |
|  | THPO | -0.753 | 4.667E-05 | 3.780E-04 |
|  | PLD4 | -0.752 | 1.159E-04 | 9.385E-04 |
|  | ASCL2 | -0.752 | 1.655E-05 | 1.341E-04 |
|  | DOC2B | -0.752 | 5.086E-07 | 4.120E-06 |
|  | CABP4 | -0.750 | 1.060E-05 | 8.590E-05 |
|  | FLRT1 | -0.749 | 4.323E-03 | 3.502E-02 |
|  | BCL11B | -0.747 | 4.220E-05 | 3.418E-04 |
|  | CLEC4F | -0.746 | 1.567E-03 | 1.269E-02 |
|  | KCNN3 | -0.744 | 1.825E-05 | 1.478E-04 |
|  | SLC17A7 | -0.742 | 6.722E-04 | 5.445E-03 |
|  | SLAMF1 | -0.741 | 1.016E-03 | 8.232E-03 |
|  | NPAS3 | -0.740 | 6.838E-04 | 5.539E-03 |
|  | GAL3ST2 | -0.738 | 3.941E-03 | 3.192E-02 |
|  | CD96 | -0.737 | 1.664E-04 | 1.348E-03 |
|  | ZAP70 | -0.735 | 1.194E-04 | 9.674E-04 |
|  | CCR4 | -0.734 | 3.616E-03 | 2.929E-02 |
|  | PKLR | -0.733 | 4.546E-03 | 3.682E-02 |
|  | AANAT | -0.732 | 2.053E-03 | 1.663E-02 |
|  | DLEC1 | -0.732 | 3.067E-04 | 2.484E-03 |
|  | MGAT3 | -0.731 | 5.025E-05 | 4.070E-04 |
|  | WFIKKN1 | -0.730 | 6.202E-05 | 5.024E-04 |
|  | CXCR6 | -0.730 | 1.714E-04 | 1.388E-03 |
|  | GRAP2 | -0.729 | 3.141E-04 | 2.544E-03 |
|  | SLA2 | -0.728 | 1.676E-04 | 1.357E-03 |
|  | DNAJC5B | -0.728 | 1.946E-03 | 1.576E-02 |
|  | DAPP1 | -0.727 | 6.331E-05 | 5.128E-04 |
|  | TAS2R10 | -0.724 | 3.342E-04 | 2.707E-03 |
|  | C16orf54 | -0.723 | 1.226E-04 | 9.931E-04 |
|  | GZMH | -0.721 | 1.360E-05 | 1.101E-04 |
|  | ZBTB32 | -0.718 | 6.375E-04 | 5.164E-03 |
|  | LTF | -0.717 | 3.946E-03 | 3.196E-02 |
|  | CDH4 | -0.717 | 1.859E-03 | 1.505E-02 |
|  | SPTBN5 | -0.716 | 5.086E-07 | 4.120E-06 |
|  | P2RY14 | -0.715 | 3.503E-04 | 2.837E-03 |
|  | CD1C | -0.714 | 3.356E-04 | 2.718E-03 |
|  | GZMK | -0.714 | 7.856E-05 | 6.363E-04 |
|  | KLRF1 | -0.713 | 6.134E-05 | 4.969E-04 |
|  | C9orf50 | -0.713 | 2.609E-03 | 2.113E-02 |
|  | CLEC12A | -0.712 | 4.956E-04 | 4.014E-03 |
|  | IL27 | -0.712 | 3.456E-04 | 2.800E-03 |
|  | SYTL1 | -0.712 | 2.344E-04 | 1.899E-03 |
|  | CLEC1B | -0.711 | 4.039E-03 | 3.272E-02 |
|  | ICOS | -0.709 | 2.203E-03 | 1.784E-02 |
|  | KCNK5 | -0.708 | 3.123E-06 | 2.530E-05 |
|  | PRG4 | -0.707 | 3.112E-03 | 2.521E-02 |
|  | ZNF683 | -0.706 | 1.214E-03 | 9.837E-03 |
|  | FAM43B | -0.705 | 3.620E-04 | 2.932E-03 |
|  | CD207 | -0.705 | 4.464E-03 | 3.616E-02 |
|  | CCL24 | -0.704 | 1.465E-03 | 1.186E-02 |
|  | SOX8 | -0.703 | 5.286E-03 | 4.282E-02 |
|  | SPN | -0.702 | 1.612E-04 | 1.305E-03 |
|  | KIF25 | -0.698 | 9.339E-04 | 7.565E-03 |
|  | CBFA2T3 | -0.697 | 1.912E-05 | 1.549E-04 |
|  | HS6ST2 | -0.697 | 7.678E-04 | 6.219E-03 |
|  | CPAMD8 | -0.697 | 3.421E-03 | 2.771E-02 |
|  | UPB1 | -0.695 | 6.258E-04 | 5.069E-03 |
|  | SLC16A8 | -0.694 | 4.529E-04 | 3.668E-03 |
|  | CD8A | -0.693 | 6.491E-05 | 5.258E-04 |
|  | SLC1A7 | -0.692 | 2.081E-03 | 1.686E-02 |
|  | ANKRD22 | -0.688 | 5.141E-04 | 4.164E-03 |
|  | IL12RB1 | -0.687 | 2.078E-05 | 1.683E-04 |
|  | RNF151 | -0.686 | 4.350E-04 | 3.524E-03 |
|  | FBXO2 | -0.684 | 3.686E-03 | 2.985E-02 |
|  | HRASLS2 | -0.684 | 5.164E-03 | 4.182E-02 |
|  | VIP | -0.683 | 1.304E-03 | 1.056E-02 |
|  | ITK | -0.680 | 1.185E-03 | 9.599E-03 |
|  | PCSK6 | -0.678 | 7.289E-04 | 5.904E-03 |
|  | CLDN20 | -0.676 | 4.641E-03 | 3.759E-02 |
|  | CD6 | -0.674 | 2.258E-04 | 1.829E-03 |
|  | TSKS | -0.673 | 5.429E-03 | 4.398E-02 |
|  | GPR17 | -0.673 | 4.999E-03 | 4.049E-02 |
|  | INHA | -0.672 | 5.586E-03 | 4.525E-02 |
|  | CCL16 | -0.671 | 4.983E-03 | 4.036E-02 |
|  | PRRG3 | -0.670 | 4.227E-04 | 3.424E-03 |
|  | GDF6 | -0.669 | 3.147E-03 | 2.549E-02 |
|  | RASGRP1 | -0.667 | 3.304E-04 | 2.676E-03 |
|  | TDRD10 | -0.667 | 3.065E-04 | 2.483E-03 |
|  | BIK | -0.664 | 4.905E-03 | 3.973E-02 |
|  | ARL5C | -0.663 | 3.198E-03 | 2.590E-02 |
|  | KCNIP2 | -0.660 | 5.505E-05 | 4.459E-04 |
|  | BMP7 | -0.660 | 1.842E-03 | 1.492E-02 |
|  | GPR171 | -0.659 | 1.363E-03 | 1.104E-02 |
|  | ABCB1 | -0.658 | 3.518E-04 | 2.850E-03 |
|  | GP1BA | -0.657 | 8.528E-04 | 6.908E-03 |
|  | CCR7 | -0.657 | 4.196E-04 | 3.399E-03 |
|  | P2RY12 | -0.656 | 1.024E-03 | 8.296E-03 |
|  | FBXL16 | -0.654 | 3.052E-03 | 2.472E-02 |
|  | BMP8A | -0.650 | 7.345E-05 | 5.950E-04 |
|  | SUCNR1 | -0.650 | 1.177E-03 | 9.532E-03 |
|  | TRPC6 | -0.649 | 6.223E-04 | 5.040E-03 |
|  | CACNG8 | -0.649 | 4.980E-03 | 4.033E-02 |
|  | NPY1R | -0.649 | 5.885E-03 | 4.767E-02 |
|  | SRMS | -0.649 | 5.599E-03 | 4.535E-02 |
|  | L1CAM | -0.647 | 5.379E-03 | 4.357E-02 |
|  | NOVA2 | -0.647 | 2.210E-07 | 1.790E-06 |
|  | GFI1 | -0.646 | 3.945E-04 | 3.195E-03 |
|  | PHOSPHO1 | -0.645 | 5.491E-03 | 4.448E-02 |
|  | TMC4 | -0.645 | 4.030E-05 | 3.265E-04 |
|  | RIC3 | -0.645 | 5.548E-03 | 4.494E-02 |
|  | GPBAR1 | -0.643 | 1.216E-03 | 9.853E-03 |
|  | VNN2 | -0.640 | 1.989E-04 | 1.611E-03 |
|  | PLCXD2 | -0.640 | 2.788E-03 | 2.258E-02 |
|  | TRAF3IP3 | -0.637 | 4.802E-04 | 3.890E-03 |
|  | FATE1 | -0.635 | 4.620E-04 | 3.743E-03 |
|  | CALML6 | -0.632 | 2.636E-04 | 2.135E-03 |
|  | KLRD1 | -0.629 | 2.128E-03 | 1.724E-02 |
|  | SLAMF6 | -0.627 | 4.485E-04 | 3.633E-03 |
|  | ATP8A1 | -0.626 | 1.280E-04 | 1.037E-03 |
|  | IL12RB2 | -0.625 | 2.700E-03 | 2.187E-02 |
|  | MADCAM1 | -0.625 | 5.930E-05 | 4.804E-04 |
|  | C1orf127 | -0.625 | 5.855E-05 | 4.743E-04 |
|  | CCDC42 | -0.625 | 2.428E-03 | 1.966E-02 |
|  | WNT10B | -0.624 | 2.211E-03 | 1.791E-02 |
|  | CYP26B1 | -0.624 | 1.519E-05 | 1.230E-04 |
|  | HPN | -0.621 | 3.931E-03 | 3.184E-02 |
|  | NAPSA | -0.621 | 1.024E-04 | 8.294E-04 |
|  | TIGD3 | -0.620 | 7.415E-05 | 6.006E-04 |
|  | VNN1 | -0.619 | 3.611E-04 | 2.925E-03 |
|  | PIK3CG | -0.617 | 2.320E-05 | 1.879E-04 |
|  | CYP17A1 | -0.614 | 3.796E-04 | 3.075E-03 |
|  | SYT15 | -0.614 | 1.462E-03 | 1.184E-02 |
|  | HRC | -0.612 | 1.085E-03 | 8.788E-03 |
|  | CCR2 | -0.611 | 7.741E-04 | 6.270E-03 |
|  | ADAMTSL2 | -0.611 | 3.552E-04 | 2.877E-03 |
|  | CX3CR1 | -0.610 | 9.070E-04 | 7.347E-03 |
|  | ADCYAP1R1 | -0.609 | 9.049E-04 | 7.330E-03 |
|  | FUT1 | -0.607 | 6.185E-06 | 5.010E-05 |
|  | TESC | -0.604 | 9.358E-06 | 7.580E-05 |
|  | KMO | -0.604 | 9.044E-04 | 7.326E-03 |
|  | PTPN7 | -0.604 | 1.640E-04 | 1.328E-03 |
|  | CR1 | -0.603 | 1.967E-03 | 1.593E-02 |
|  | CD5 | -0.602 | 5.979E-04 | 4.843E-03 |
|  | P2RY8 | -0.602 | 1.864E-06 | 1.510E-05 |
|  | TFCP2L1 | -0.601 | 2.707E-03 | 2.192E-02 |
|  | CD244 | -0.601 | 2.542E-03 | 2.059E-02 |
|  | CETP | -0.599 | 3.230E-05 | 2.616E-04 |
|  | KCNQ3 | -0.597 | 3.716E-03 | 3.010E-02 |
|  | CMYA5 | -0.595 | 2.289E-03 | 1.854E-02 |
|  | MCOLN2 | -0.594 | 9.360E-04 | 7.582E-03 |
|  | IL18RAP | -0.592 | 9.907E-04 | 8.025E-03 |
|  | DISC1 | -0.591 | 1.309E-07 | 1.060E-06 |
|  | MAP4K1 | -0.590 | 1.513E-05 | 1.225E-04 |
|  | DERL3 | -0.589 | 3.365E-04 | 2.726E-03 |
|  | FGD2 | -0.589 | 3.194E-05 | 2.587E-04 |
|  | PLCG2 | -0.589 | 2.667E-08 | 2.160E-07 |
|  | CARD11 | -0.588 | 1.267E-04 | 1.027E-03 |
|  | CLEC10A | -0.587 | 1.139E-04 | 9.230E-04 |
|  | EPS8L1 | -0.586 | 1.153E-03 | 9.338E-03 |
|  | C17orf47 | -0.585 | 5.403E-03 | 4.376E-02 |
|  | CDH6 | -0.583 | 2.791E-03 | 2.261E-02 |
|  | XCL2 | -0.582 | 1.376E-03 | 1.115E-02 |
|  | DKK1 | -0.581 | 2.081E-03 | 1.685E-02 |
|  | SPATC1 | -0.581 | 6.777E-04 | 5.489E-03 |
|  | SH2D1B | -0.581 | 3.015E-03 | 2.443E-02 |
|  | CXCL9 | -0.580 | 1.623E-04 | 1.315E-03 |
|  | TMEM30B | -0.578 | 1.008E-03 | 8.163E-03 |
|  | S100A14 | -0.576 | 5.959E-03 | 4.826E-02 |
|  | PPP1R16B | -0.576 | 4.889E-06 | 3.960E-05 |
|  | CCL19 | -0.576 | 2.931E-03 | 2.374E-02 |
|  | CACNA1F | -0.574 | 4.083E-04 | 3.307E-03 |
|  | AVPR2 | -0.572 | 1.108E-03 | 8.978E-03 |
|  | ATP1B2 | -0.572 | 6.828E-04 | 5.531E-03 |
|  | EPB41L4B | -0.568 | 1.990E-03 | 1.612E-02 |
|  | FOXP3 | -0.568 | 1.406E-04 | 1.139E-03 |
|  | SIT1 | -0.568 | 5.017E-04 | 4.064E-03 |
|  | PLA2G2A | -0.567 | 4.153E-03 | 3.364E-02 |
|  | NGEF | -0.566 | 1.972E-03 | 1.598E-02 |
|  | XG | -0.565 | 1.100E-03 | 8.911E-03 |
|  | PSD4 | -0.564 | 8.494E-07 | 6.880E-06 |
|  | UNC5A | -0.564 | 1.155E-03 | 9.355E-03 |
|  | CRHR2 | -0.562 | 3.974E-03 | 3.219E-02 |
|  | PDZD3 | -0.562 | 1.410E-03 | 1.142E-02 |
|  | SLC5A9 | -0.561 | 1.518E-03 | 1.230E-02 |
|  | PLA2G5 | -0.561 | 3.289E-03 | 2.664E-02 |
|  | MMP25 | -0.561 | 9.117E-04 | 7.385E-03 |
|  | MED12L | -0.560 | 2.195E-03 | 1.778E-02 |
|  | ZNF396 | -0.560 | 4.679E-07 | 3.790E-06 |
|  | FGD3 | -0.559 | 1.687E-05 | 1.366E-04 |
|  | CD2 | -0.559 | 1.490E-04 | 1.207E-03 |
|  | FAM78B | -0.557 | 2.448E-04 | 1.983E-03 |
|  | CD79B | -0.556 | 1.905E-05 | 1.543E-04 |
|  | PARP15 | -0.554 | 2.437E-03 | 1.974E-02 |
|  | RASGEF1A | -0.553 | 5.866E-03 | 4.751E-02 |
|  | ZNF423 | -0.553 | 1.633E-04 | 1.323E-03 |
|  | HLA-DOB | -0.551 | 1.034E-03 | 8.373E-03 |
|  | CCR5 | -0.551 | 2.385E-04 | 1.932E-03 |
|  | SCN9A | -0.550 | 3.779E-03 | 3.061E-02 |
|  | ITGAL | -0.549 | 6.744E-05 | 5.463E-04 |
|  | PFKFB1 | -0.549 | 8.180E-05 | 6.626E-04 |
|  | CTRC | -0.547 | 4.714E-03 | 3.818E-02 |
|  | DNASE1L3 | -0.547 | 3.299E-03 | 2.672E-02 |
|  | TAS2R4 | -0.546 | 5.036E-04 | 4.079E-03 |
|  | SAMD3 | -0.546 | 2.536E-03 | 2.054E-02 |
|  | IRF4 | -0.545 | 3.789E-03 | 3.069E-02 |
|  | MFRP | -0.542 | 3.005E-03 | 2.434E-02 |
|  | XCL1 | -0.540 | 2.335E-03 | 1.891E-02 |
|  | SIDT1 | -0.540 | 7.104E-04 | 5.754E-03 |
|  | ICA1 | -0.540 | 1.716E-06 | 1.390E-05 |
|  | CD3E | -0.539 | 3.390E-04 | 2.746E-03 |
|  | SARDH | -0.539 | 3.009E-03 | 2.437E-02 |
|  | PLAC8 | -0.538 | 2.232E-03 | 1.808E-02 |
|  | ABCC11 | -0.537 | 5.535E-03 | 4.483E-02 |
|  | IL21R | -0.536 | 2.907E-03 | 2.355E-02 |
|  | GZMM | -0.533 | 1.502E-04 | 1.217E-03 |
|  | DTX4 | -0.533 | 2.466E-05 | 1.997E-04 |
|  | CD200R1 | -0.532 | 3.445E-04 | 2.790E-03 |
|  | AVPR1A | -0.531 | 7.382E-04 | 5.979E-03 |
|  | MEOX1 | -0.529 | 1.313E-03 | 1.064E-02 |
|  | ABCA8 | -0.529 | 3.052E-03 | 2.472E-02 |
|  | LILRA2 | -0.529 | 1.049E-03 | 8.500E-03 |
|  | TFEC | -0.528 | 3.589E-04 | 2.907E-03 |
|  | TRIM17 | -0.526 | 5.716E-03 | 4.630E-02 |
|  | GRHL1 | -0.526 | 4.456E-03 | 3.610E-02 |
|  | NR5A2 | -0.526 | 6.190E-04 | 5.014E-03 |
|  | ATF7IP2 | -0.526 | 1.418E-04 | 1.149E-03 |
|  | TLR8 | -0.525 | 5.986E-04 | 4.848E-03 |
|  | PDE6C | -0.525 | 4.190E-05 | 3.394E-04 |
|  | AKR7A3 | -0.525 | 1.942E-03 | 1.573E-02 |
|  | SLC16A4 | -0.524 | 5.105E-04 | 4.135E-03 |
|  | GRAP | -0.522 | 2.171E-04 | 1.759E-03 |
|  | SPATA18 | -0.522 | 2.774E-03 | 2.247E-02 |
|  | ACR | -0.521 | 1.217E-03 | 9.855E-03 |
|  | BMP6 | -0.519 | 3.189E-04 | 2.583E-03 |
|  | OBSCN | -0.518 | 5.753E-03 | 4.660E-02 |
|  | ADCY4 | -0.518 | 5.086E-07 | 4.120E-06 |
|  | CRLF1 | -0.517 | 5.483E-03 | 4.441E-02 |
|  | NPPA | -0.516 | 1.812E-03 | 1.468E-02 |
|  | POMC | -0.514 | 1.613E-03 | 1.306E-02 |
|  | NGFR | -0.508 | 6.597E-04 | 5.344E-03 |
|  | DOCK8 | -0.508 | 3.255E-05 | 2.637E-04 |
|  | ADAM28 | -0.507 | 1.451E-03 | 1.175E-02 |
|  | ZMYND12 | -0.507 | 3.848E-05 | 3.117E-04 |
|  | PMFBP1 | -0.506 | 7.954E-04 | 6.443E-03 |
|  | GPR141 | -0.505 | 1.745E-03 | 1.413E-02 |
|  | RHPN1 | -0.504 | 8.870E-05 | 7.185E-04 |
|  | TMC8 | -0.502 | 3.391E-05 | 2.747E-04 |
|  | DENND1C | -0.502 | 1.369E-04 | 1.109E-03 |
|  | SP140 | -0.501 | 3.252E-03 | 2.634E-02 |
|  | TREX2 | -0.501 | 2.236E-04 | 1.811E-03 |
|  | ARHGAP27 | -0.500 | 1.790E-08 | 1.450E-07 |
|  | TMPRSS9 | -0.500 | 2.769E-03 | 2.243E-02 |

*FC* Fold change; *FDR* False Discovery Rate
